# Supplementary material for: Dissecting Fc signatures of protection in neonates following maternal influenza vaccination in a placebo-controlled trial
Source: Cell Rep. 2022 Feb 8;38(6):110337. doi: 10.1016/j.celrep.2022.110337 (PMC9026287; doi:10.1016/j.celrep.2022.110337)
Supplement: Document S1. Figures S1–S5 and Tables S1 and S2 [file mmc1.pdf]

Cell Reports, Volume *38*

Supplemental information

Dissecting Fc signatures of protection in neonates following maternal influenza vaccination in a placebo-controlled trial

Carolyn M. Boudreau, John S. Burke IV, Kiel D. Shuey, Caitlin Wolf, Joanne Katz, James Tielsch, Subarna Khatry, Steven C. LeClerq, Janet A. Englund, Helen Y. Chu, and Galit Alter

Figure S1. Vaccination boosts maternal and fetal antibodies, but not transfer efficacy, Related to Figure 2.

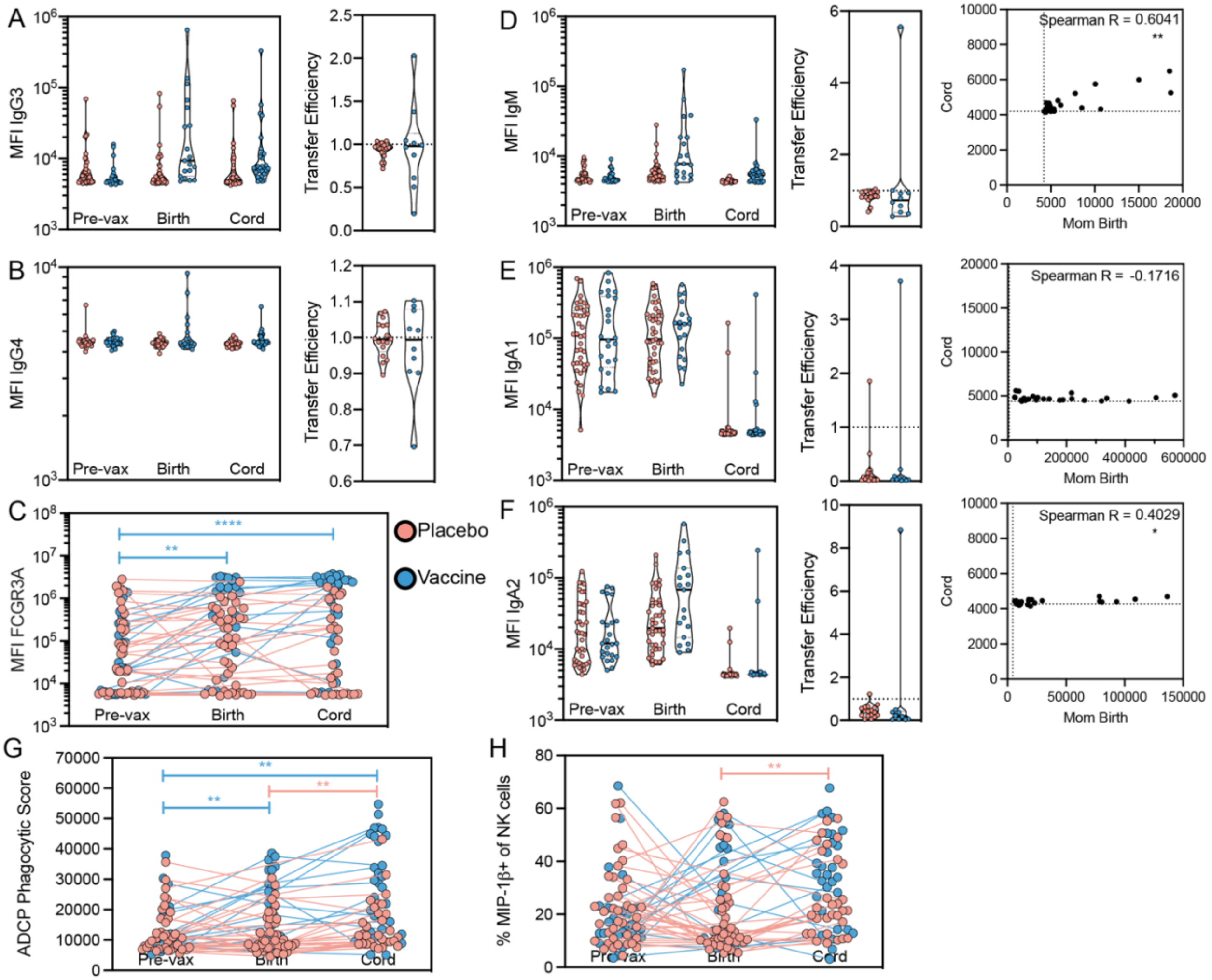

Paired violin plots show levels of H1 A/California/07/2009-specific antibodies pre-vaccination in maternal circulation, at the time of birth in maternal circulation, and in fetal circulation in the cord blood at the time of birth and the transfer efficiency of each type of antibody across the placenta at the time of birth. Each dot represents an individual and violins show the distribution of the group. Red dots represent pairs where the mother received placebo and blue dots represent pairs where the mother received influenza vaccination. Violin plots show transfer efficiencies calculated as the ratio between cord blood antibodies and maternal antibodies at time of birth. (**A**) H1-specific IgG3 by Luminex MFI; (**B**) H1-specific IgG4 by Luminex MFI; (**D**) H1-specific IgM by Luminex MFI; (**E**) H1-specific IgA1 by Luminex MFI; (**F**) H1-specific IgA2 by Luminex MFI. Dot plots (**D-F**) show correlation between H1 A/California/07/2009-specific isotype MFIs in maternal circulation at the time of birth (X axis) and in cord blood at the time of birth (Y axis). Each dot represents a matched mother-child pair. Dotted lines represent

the lower limit of detection of the assay. Spearman R values are reported with significance. Colored dot plots show change over time for connected maternal:fetal dyads. (**C**) H1-specific FCGR3A by Luminex MFI; (**G**) Phagocytosis by monocytes of H1-coated immune complexed beads; (**H**) MIP-1b expression by H1-specific antibody-stimulated NK cells. Significance was calculated by mixed effects analysis with Sidak's multiple comparisons test or Mann-Whitney U test as appropriate, * $p < 0.05$, ** $p < 0.01$, *** $p < 0.001$, **** $p < 0.0001$.

Figure S2. Vaccination boosts maternal and fetal antibodies specific to H3, Related to Figure 2

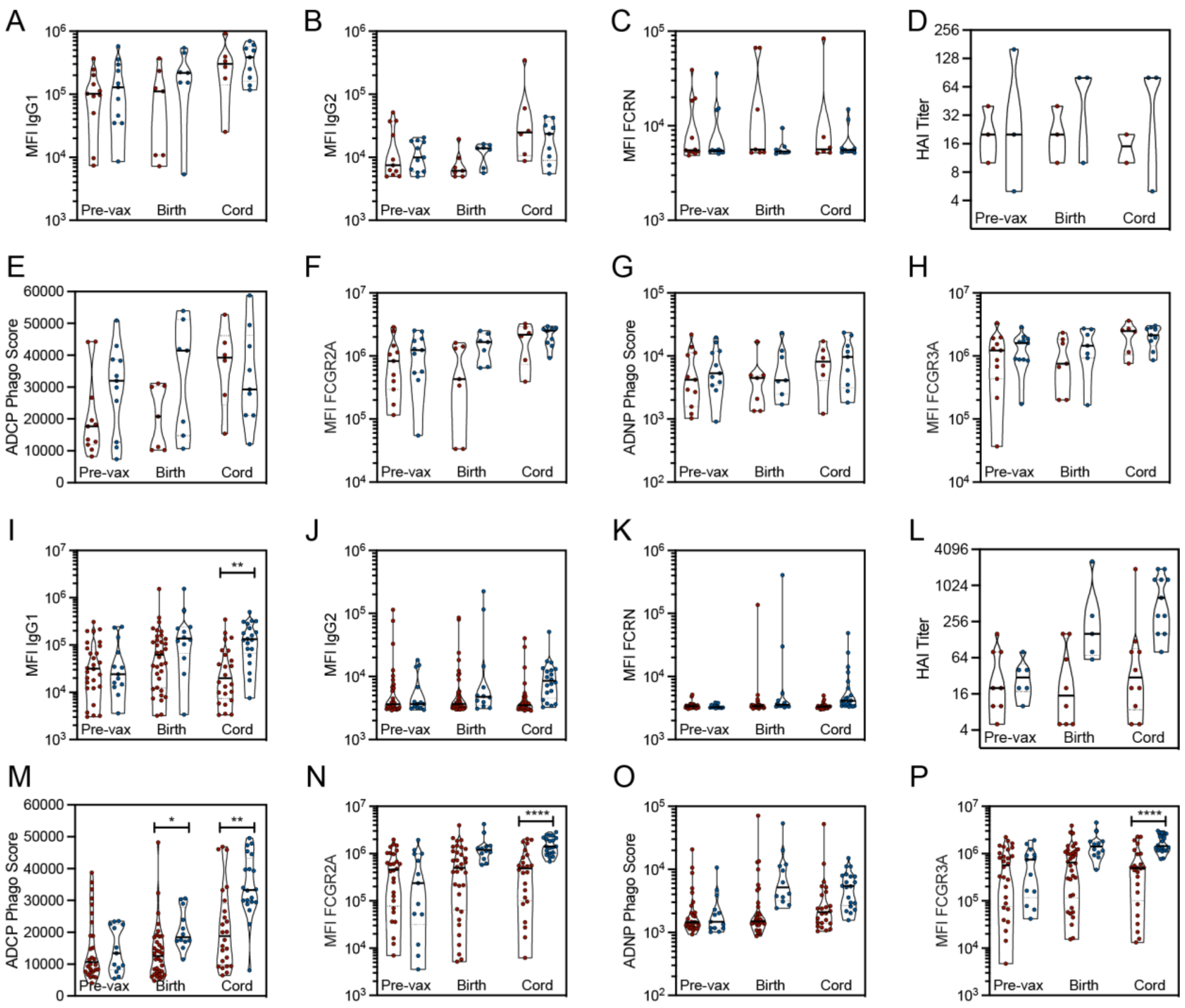

Paired violin plots show levels of (**A-H**) H3 A/Perth/16/2009-specific or (**I-P**) H3 A/Victoria/361/2011-specific antibodies pre-vaccination in maternal circulation, at the time of birth in maternal circulation, and in fetal circulation in the cord blood at the time of birth and the transfer efficiency of each type of antibody across the placenta at the time of birth. Each dot represents an individual and violins show the distribution of the group. Red dots represent pairs where the mother received placebo and blue dots represent pairs where the mother received influenza vaccination. (**A,I**) IgG1 by Luminex MFI; (**B,J**) IgG2 by Luminex MFI; (**C,K**) FCRN binding by Luminex MFI; (**D,L**) HAI titers; (**E,M**) cellular phagocytosis; (**F,N**) FCGR2A binding by Luminex MFI; (**G,O**) neutrophil phagocytosis; (**H,P**) FCGR3A binding by Luminex MFI. For (**A-C, E-H**) H3 A/Perth/16/2009-specific responses: pre-vax placebo n = 11, pre-vax vaccine n = 11,

time of birth placebo n = 7, time of birth vaccine n = 7, cord placebo n = 6, cord vaccine n = 9. For (**D**) H3 A/Perth/16/2009-specific HAI: pre-vax placebo n = 3, pre-vax vaccine n = 3, time of birth placebo n = 3, time of birth vaccine n = 3, cord placebo n = 2, cord vaccine n = 3. For (**I-K, M-P**) H3 A/Victoria/361/2011-specific responses: pre-vax placebo n = 28, pre-vax vaccine n = 13, time of birth placebo n = 34, time of birth vaccine n = 12, cord placebo n = 24, cord vaccine n = 20. For (**L**) H3 A/Victoria/361/2011-specific HAI: pre-vax placebo n = 8, pre-vax vaccine n = 6, time of birth placebo n = 8, time of birth vaccine n = 5, cord placebo n = 10, cord vaccine n = 11.

Figure S3. HAI is not a key feature separating placebo recipients and vaccinees but correlates with some key features, Related to Figure 3

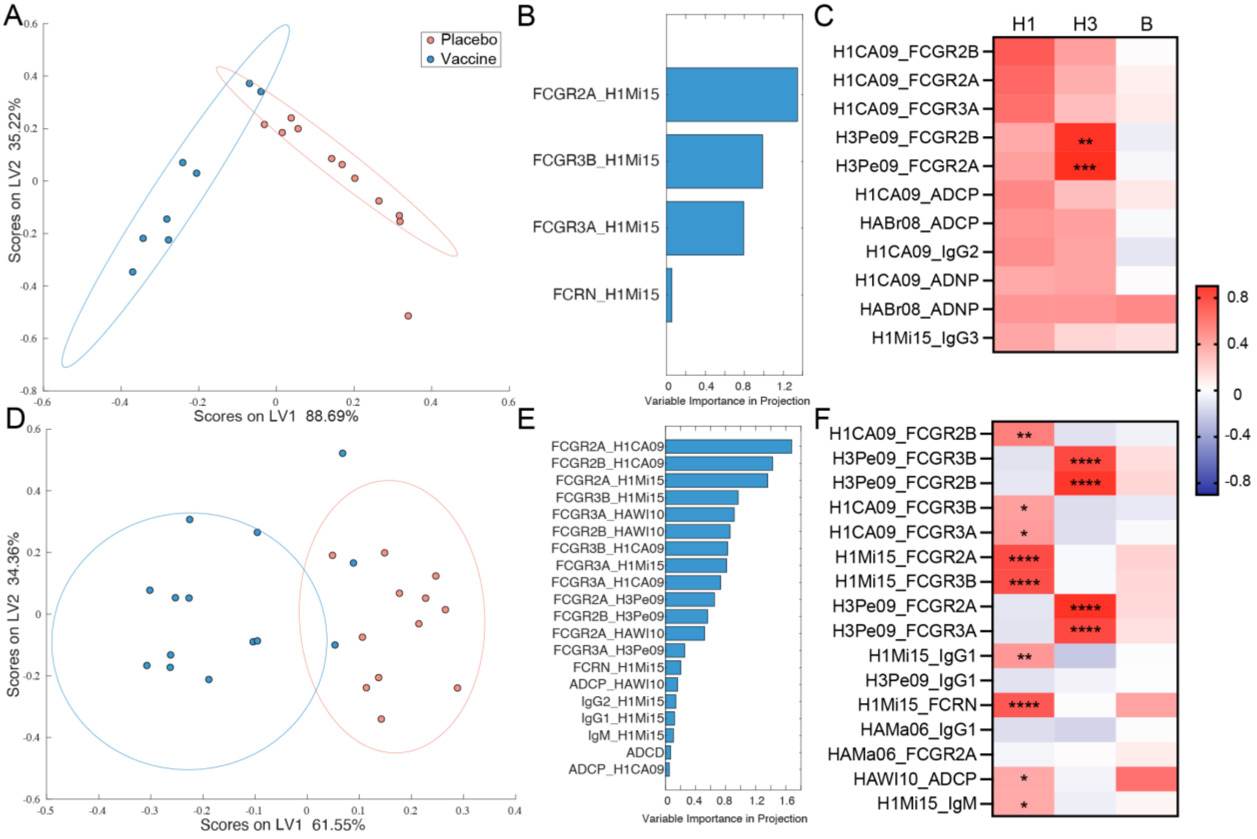

Dot plots show PLSDA scores along latent variable 1 (LV1) and latent variable 2 (LV2) for maternal samples at the time of birth (**A**, p < 0.001 compared to permuted label model) and cord blood samples (**D**, p < 0.01 compared to permuted label model). PLSDA was modeled using LASSO-selected features out of the total pool of measured humoral features, including HAI. LV percentages represent the amount of total variance between samples captured by that LV. Bar plots show VIP scores for the LASSO-selected features separating vaccinees from placebo recipients for maternal samples at the time of birth (**B**) and cord blood samples (**E**). VIP scores reflect the contribution of a feature across all latent variables. Correlation matrices show Spearman correlation R values calculated pairwise between HAI titers and LASSO-selected features for samples taken from maternal circulation (**C**) and cord blood (**F**) at the time of birth. Stars indicate Bonferroni-corrected p values: * < 0.05, ** < 0.01, *** < 0.001, **** <0.0001.

Figure S4. HAI titers do not significantly correlate with key features separating infected and uninfected infants, Related to Figure 4.

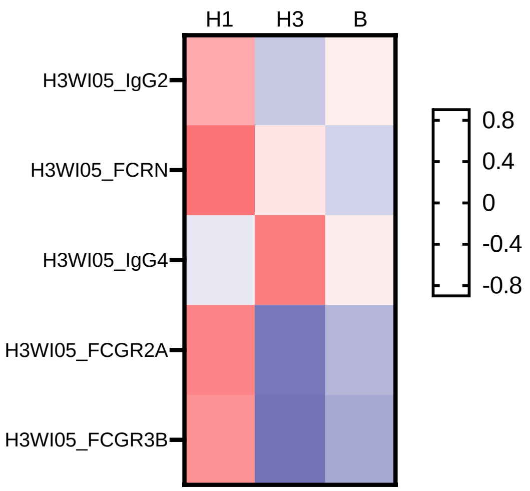

Correlation matrix shows Spearman correlation R values calculated pairwise between HAI titers and LASSO-selected features for samples cord blood at the time of birth for infants who went on to become infected with H3N2 influenza or remained uninfected throughout the trial. No correlations were significant when corrected for multiple comparisons.

Figure S5. Vaccine-induced humoral features are predictive of protection from influenza infection in infants, Related to Figure 6.

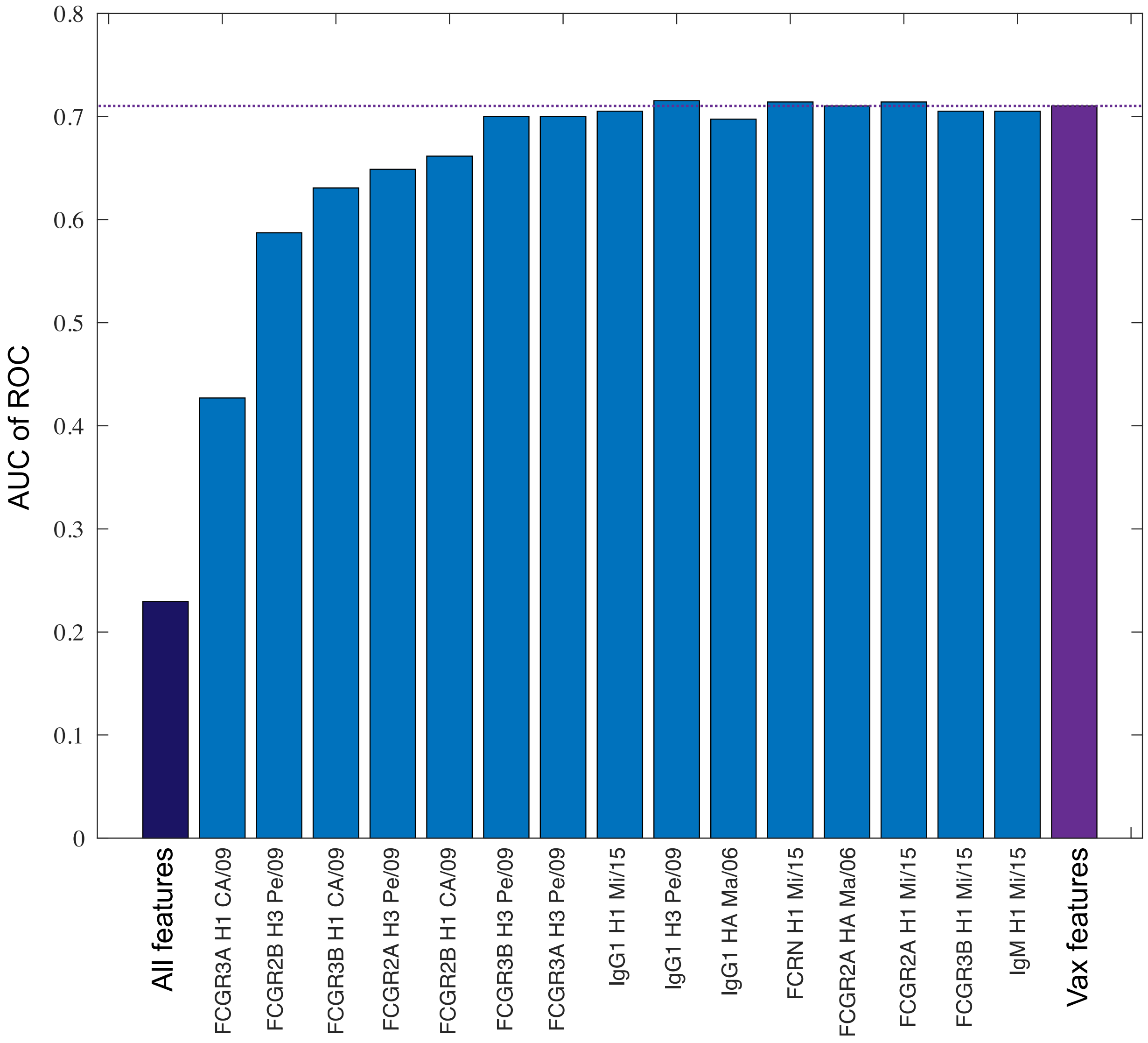

Bar plot shows the area under the curve for ROC of the PLSDA separating H3-infected infants from uninfected infants. Dark blue bar (left) shows AUC of ROC for all measured humoral features (as shown in Figure 6). Purple bar (right) shows AUC of ROC for vaccine LASSO-selected humoral features (as shown in Figure 6). Blue bars in between show sequential addition of individual features; leftmost blue bar shows only FCGR3A H1 CA/09, then each sequential feature is added moving from left to right.

Table S1. Demographic information about study participants, Related to STAR Methods

	Placebo	Vaccine	Control	Case
**Demographic Data Available**	60	49	77	32
**Missing Demographic Data**	1	0	0	1
**Male infants**	16.7% (10)	26.5% (13)	23.4% (18)	15.6% (5)
**Preterm infants**	28.3% (17)	20.4% (10)	23.4% (18)	28.1% (9)
**Vaccine Strains**				
A/CA/07/2009, A/Perth/16/2009, B/Brisbane/60/2008	23.3% (14)	30.6% (15)	28.6% (22)	21.9% (7)
A/CA/07/2009, A/Victoria/361/2011, B/Wisconsin/1/2010	76.7% (46)	69.4% (34)	71.4% (55)	78.1% (25)
**Vaccination Season**				
Dec-Feb	55% (33)	30.6% (15)	41.6% (32)	50% (16)
Mar-May	21.7% (13)	34.7% (17)	27.3% (21)	28.1% (9)
Jun-Aug	1.7% (1)	4.1% (2)	4.9% (3)	0% (0)
Sep-Nov	21.7% (13)	30.6% (15)	27.3% (21)	21.9% (7)
**Birth Season**				
Dec-Feb	18.3% (11)	40.8% (20)	32.5% (25)	18.8% (6)
Mar-May	48.3% (29)	14.3% (7)	29.9% (23)	40.6% (13)
Jun-Aug	31.7% (19)	32.7% (16)	32.5% (25)	31.3% (10)
Sep-Nov	1.7% (1)	12.2% (6)	5.2% (4)	9.4% (3)
**Approx. Gestational Age at Vaccination**				
1st Trimester	3.3% (2)	6.1% (3)	5.2% (4)	3.1% (1)
2nd Trimester	76.7% (46)	61.2% (30)	66.2% (51)	78.1% (25)
3rd Trimester	20% (12)	32.7% (16)	28.6% (22)	18.8% (6)

Table S2. Significance of covariates to vaccination across placebo and vaccine recipient pairs, related to Figure 2.

Measurement	Significance of Covariate (p)	Bonferroni corrected p	
IgG1	6.45E-06	7.09E-05	****
IgG2	1.29E-04	0.0014	**
FCRN	0.0465	0.5111	ns
ADCP	5.78E-05	6.36E-04	***
FCGR2A	9.32E-10	1.03E-08	****
ADNP	0.0013	0.0138	*
ADCD	7.53E-05	8.29E-04	***
ADNKA CD107a	2.91E-05	3.20E-04	***
ADNKA IFN-y	3.09E-04	0.0034	**
ADNKA MIP-1b	2.17E-04	0.0024	**
FCGR3A	1.41E-06	1.55E-05	****
