# Supplementary material for: Dissecting Fc signatures of protection in neonates following maternal influenza vaccination in a placebo-controlled trial
Source: Cell Rep. 2022 Feb 8;38(6):110337. doi: 10.1016/j.celrep.2022.110337 (PMC9026287; doi:10.1016/j.celrep.2022.110337)
Supplement: Document S2. Article plus supplemental information [file mmc9.pdf]

Article

Cell Reports

Dissecting Fc signatures of protection in neonates following maternal influenza vaccination in a placebo-controlled trial

Graphical abstract

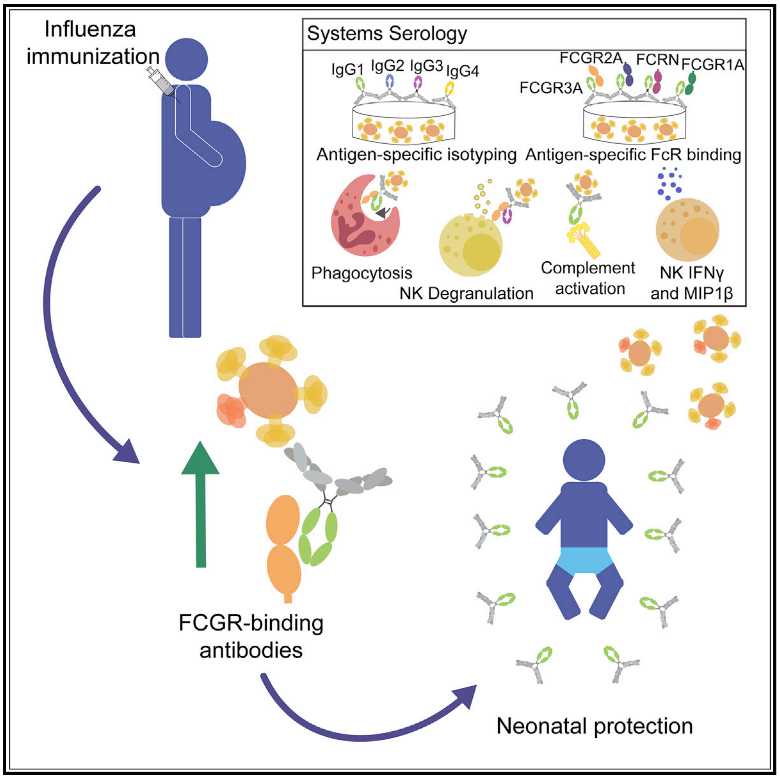

Authors

Carolyn M. Boudreau, John S. Burke IV, Kiel D. Shuey, ..., Janet A. Englund, Helen Y. Chu, Galit Alter

Correspondence

janet.englund@seattlechildrens.org (J.A.E.),
helenchu@uw.edu (H.Y.C.),
galter@mgh.harvard.edu (G.A.)

In brief

Boudreau et al. characterize the antibodies induced by influenza vaccination during pregnancy and describe which of these antibodies are transferred to the infant. They show that the antibodies arising after vaccination provide protection from influenza to infants during the first months of life.

Highlights

- Maternal influenza vaccination induces innate immune-functional antibodies
- These antibodies are transferred across the placenta to the developing fetus
- Antibodies that bind Fc γ receptors protect infants from influenza
- Maternal vaccination induces antibody qualities linked to protection in infants

Boudreau et al., 2022, Cell Reports *38*, 110337
February 8, 2022 © 2022 The Authors.
https://doi.org/10.1016/j.celrep.2022.110337

Cell Reports

CellPress
OPEN ACCESS

Article

Dissecting Fc signatures of protection in neonates following maternal influenza vaccination in a placebo-controlled trial

Carolyn M. Boudreau,[1,2] John S. Burke IV,[1] Kiel D. Shuey,[3] Caitlin Wolf,[3] Joanne Katz,[4] James Tielsch,[5] Subarna Khatry,[6] Steven C. LeClerq,[4,6] Janet A. Englund,[7,*] Helen Y. Chu,[3,*] and Galit Alter[1,8,*]
[1]Ragon Institute of MGH, MIT, and Harvard, Cambridge, MA, USA
[2]PhD Program in Virology, Harvard University, Cambridge, MA, USA
[3]Department of Medicine, University of Washington, Seattle, WA, USA
[4]Department of International Health, Johns Hopkins Bloomberg School of Public Health, Baltimore, MD, USA
[5]Department of Global Health, Milken Institute School of Public Health, George Washington University, Washington, DC, USA
[6]Nepal Nutrition Intervention Project, Sarlahi, Kathmandu, Nepal
[7]Department of Pediatrics, Seattle Children's Research Institute and University of Washington, Seattle, WA, USA
[8]Lead contact
*Correspondence: janet.englund@seattlechildrens.org (J.A.E.), helenchu@uw.edu (H.Y.C.), galter@mgh.harvard.edu (G.A.)
https://doi.org/10.1016/j.celrep.2022.110337

SUMMARY

Influenza is an important cause of illness and morbidity for infants. Seasonal influenza vaccination during pregnancy aims to provide protection to mothers, but it can also provide immunity to infants. The precise influence of maternal vaccination on immunity in infants and how vaccine-elicited antibodies provide protection in some but not all infants is incompletely understood. We comprehensively profiled the transfer of functional antibodies and defined humoral factors contributing to immunity against influenza in a clinical trial of maternal influenza vaccination. Influenza-specific antibody subclass levels, Fc ɣ receptor (FCGR) binding levels, and antibody-dependent innate immune functions were all profiled in the mothers during pregnancy and at birth, as well as in cord blood. Vaccination increased influenza-specific antibody levels, antibody binding to FCGR, and specific antibody-dependent innate immune functions in both maternal and cord blood, with FCGR binding most enhanced via vaccination. Influenza-specific FCGR binding levels were lower in cord blood of infants who subsequently developed influenza infection. Collectively these data suggest that in addition to increased antibody amounts, the selective transfer of FCGR-binding antibodies contributes to the protective immune response in infants against influenza.

INTRODUCTION

Influenza is a major cause of respiratory disease and hospitalization in children under 5 years of age globally (El Guerche-Séblain et al., 2019; Nair et al., 2011; Ortiz et al., 2011). Moreover, infants and young children without underlying medical conditions are hospitalized for influenza-associated illness at higher rates than adults with high-risk conditions, including the elderly (Tielsch et al., 2015; Wang et al., 2020). These data have revealed the critical need for global vaccine campaigns to protect high-risk groups, including young children, elderly, and pregnant people, leading to the recommendation of year-round influenza vaccination during pregnancy to protect both the mother and child (World Health Organization, 2012), although vaccination remains available only seasonally in much of the world.

While pregnancy is not associated with an increased risk of influenza virus infection, pregnancy is associated with an increased risk of hospitalization and adverse outcomes following an influenza infection, particularly during the third trimester (Neuzil et al., 1998; Omer et al., 2015). Specifically, influenza infection during pregnancy is associated with an increased risk of low infant birthweight, preterm birth, neonatal and maternal ICU admission, and even infant death (Doyle et al., 2013). Influenza vaccination during pregnancy decreases rates of respiratory illness in both the mother and the infant (Dabrera et al., 2014; Jarvis et al., 2020; Katz et al., 2018; Nunes and Madhi, 2018; Tapia et al., 2016; Zaman et al., 2008), with efficacy rates among mothers estimated at 31%–70% and efficacy rates in infants estimated at 30%–63% across multiple randomized, controlled trials (Omer, 2017), clearly highlighting the public health impact of influenza vaccination during pregnancy.

Influenza vaccination during pregnancy has been linked to elevated vaccine-specific antibody concentrations in the cord blood that persist in the neonate for at least 2 months post-partum (Englund et al., 1993; Tapia et al., 2016; Zhong et al., 2019). However, the mechanism(s) through which these transferred antibodies afford protection against influenza remains incompletely understood. The current standard for evaluating influenza

This is an open access article under the CC BY license (http://creativecommons.org/licenses/by/4.0/).

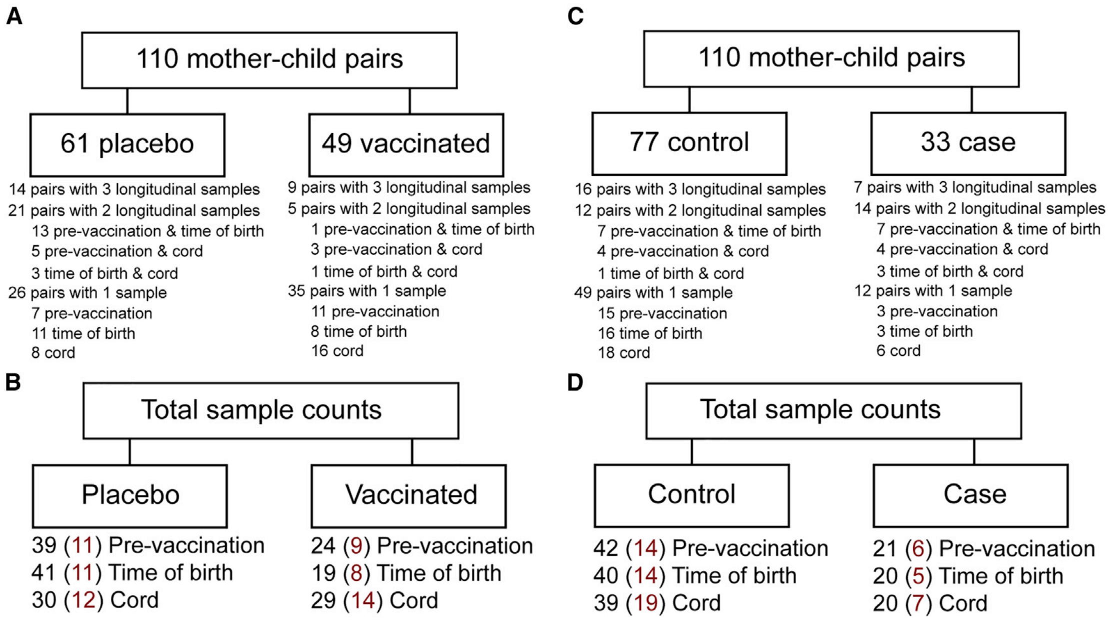

Figure 1. Schematic of samples included in this study

(A–D) The schematic (A) shows dyads with numbers of longitudinal samples across placebo and vaccinated groups. The schematic (B) shows the number of samples at each time point in each group. Numbers in red indicate samples for which HAI data is available. The schematic (C) shows dyads with number of longitudinal samples across control (uninfected infants) and case (infants who became infected). Schematic (D) shows number of samples at each time point in each group. Numbers in red indicate samples for which HAI data is available.

vaccination immunogenicity is the hemagglutination inhibition (HAI) assay (Ohmit et al., 2011), which is a proxy for viral neutralization. While neutralizing antibodies that opsonize the virus and prevent infection are undoubtedly important for protection, increasing evidence points to additional antibody-induced functions in protection from influenza infection (Boudreau and Alter, 2019). Extra-neutralizing antibody functions mediate protection against influenza in animal models (Dilillo et al., 2014; DiLillo et al., 2016; He et al., 2017; Huber et al., 2001; Jegaskanda et al., 2017; Mullarkey et al., 2016; O'Brien et al., 2011; Rattan et al., 2017; Terajima et al., 2015; Wong et al., 2017) and have been observed following infection and vaccination in humans (Co et al., 2012; Jegaskanda et al., 2013a, 2017; Vanderven et al., 2020). Specifically, the ability of influenza-specific antibodies to drive Fc ɣ receptor (FCGR)-dependent functions, namely antibody-dependent cellular phagocytosis (ADCP) (Jegaskanda, 2018; Mullarkey et al., 2016), neutrophil production of reactive oxygen species (Mullarkey et al., 2016), antibody-dependent cellular cytotoxicity (Huber et al., 2001), and antibody-dependent complement deposition (ADCD) (Jegaskanda, 2018; Kopf et al., 2002; O'Brien et al., 2011; Rattan et al., 2017), have been linked to protection from influenza infection and disease. Whether influenza vaccination during pregnancy can induce functional antibodies, and whether these antibodies are transferred to the fetus and provide protection in infants, remains unknown.

During pregnancy, antibodies are actively transported across the placenta to the fetus, where they enter fetal circulation and provide protection during the first months of life (Doyle et al., 2013; Englund et al., 1993; Tapia et al., 2016). Neonatal Fc receptor (FcRn) preferentially transfers IgG1 antibodies across the placenta (Akbulut et al., 2012; Clements et al., 2020; Costa-Carvalho et al., 1996; Hashira et al., 2000; Stapleton et al., 2011). Recent studies have further indicated that the placenta may selectively transfer subsets of IgG1 antibodies with enhanced NK cell function to provide protection to infants in early life (Jennewein et al., 2019). Thus, we aimed to define the influence of influenza vaccination on tuning influenza-specific immunity during pregnancy, to define how vaccination impacts antibody transfer to neonates, and ultimately to probe the specific patterns of antibodies that track with protection in early life. Using systems serology (Chung et al., 2015), we profiled the influenza-specific immune response among a cohort of pregnant women in rural Nepal (Figure 1), where women were enrolled in a randomized clinical trial of influenza vaccination and were administered vaccine or placebo between 17 and 34 weeks of gestation (Omer et al., 2015; Steinhoff et al., 2017). Influenza-specific antibody profiles were then comprehensively assessed in the maternal and cord blood among infants with and without influenza infection from birth to six months (Omer et al., 2015).

RESULTS

Vaccination boosts antibody levels, FCGR binding, and functionality

Previous studies have demonstrated that influenza vaccination during pregnancy results in a significant augmentation of influenza-specific antibody titers (Takeda et al., 2015), as well as serum levels of hemagglutination inhibition (HAI) (Katz et al.,

2018) (Figure S1A) and neutralization (Kay et al., 2015). However, it remains unclear whether vaccination also is able to improve or alter antibody functionality. Thus, we applied systems serology (Chung et al., 2015) to comprehensively profile influenza-specific antibodies in vaccinated and unvaccinated mothers and their infants. Given that there was a change in vaccine strains over the study period, the humoral immune response was dissected across all hemagglutinin (HA) strains included in the trivalent vaccine during the study period. For analyses comparing vaccination to placebo (Figures 2A and 2B), we focused on H1-specific antibodies, as the influenza A H1N1 strain did not change during the study period, giving our analysis increased power and removing strain variability as a confounding factor.

No differences were observed in H1-specific IgG1 levels across vaccinated and unvaccinated mothers prior to vaccination, while increased H1-specific IgG1 levels were observed in vaccinated compared to unvaccinated mothers at the time of birth (Figure 2A). This maternal antibody elevation resulted in increased H1-specific IgG1 levels in the cord blood as well. A similar trend was observed for H1-specific IgG2 levels (Figure 2B), whereas no significant change following vaccination was observed for any other antibody subclass (Figures S1A and S1B) or isotype (Figures S1D–S1F). Little to no transfer of IgA or IgM was observed, as expected (Figures S1D–S1F).

IgG transfer across the placenta depends on FcRn (Simister and Mostov, 1989; Simister and Rees, 1985). To determine whether vaccination altered FcRn binding, thereby improving antibody transfer, we next examined H1-specific FcRn binding. H1-specific antibodies in sera from vaccinees bound at higher levels to FcRn compared to the placebo serum (Figure 2C); however, only a subset of vaccinees (n = 7) displayed this increase, while others remained close to baseline levels. Similarly, enhanced binding of H1-specific antibodies to FcRn was also observed in a subset of cord blood samples (n = 10) from the vaccine arm (Figure 2C). No demographic data correlated with these distinct antibody responses, including infant sex, influenza outcome, or infant birth month. This study was not powered to determine drivers of non-responsiveness, although we observed an enrichment of term births in responders, and pre-term births in the non-responders. This elevation in FcRn binding is likely at least partially attributable to the presence of higher influenza-specific IgG levels after vaccination (Figures 2A and 2C). These findings indicate that vaccination both qualitatively and quantitatively changes the antibody response, enhancing the placental transfer of antibodies.

In addition to enhanced binding to FcRn, vaccination also improved neutralizing antibody titers, antibody-dependent innate immune functions, and binding to activating FCGRs. Specifically, HAI titers, the major correlate of vaccine-induced protection against influenza infection, were significantly increased in both maternal and cord circulation following vaccination (Figure 2D). Additionally, ADCP by monocytes was increased following vaccination; this increase was transferred to neonates (Figure 2E), as was binding to the FCGR most commonly associated with phagocytosis, FCGR2A (Figure 2F). Similarly, both increases in antibody-dependent neutrophil phagocytosis and ADCD were elevated in maternal and cord blood following vaccination (Figures 2G and 2H). Interestingly, levels of H1-specific antibodies capable of inducing NK cell cytotoxicity (measured by CD107a expression) and cytokine release (measured by IFN-ɣ) were augmented in cord blood by vaccination, but vaccination had a limited effect on altering the levels of NK cell chemokine release-inducing antibodies (Figures 2I–2K), corroborating previous data pointing to preferential transfer of NK cell activating antibodies (Jennewein et al., 2019). Interestingly, FCGR3A-binding antibodies increased spontaneously during pregnancy for some individuals (Figures 2L and S1C), pointing to a shift in the quality of H1-specific antibodies during pregnancy, augmented by vaccination, poised for placental transfer.

To directly compare placental transfer efficiencies across all antibody features, the ratio between cord and maternal antibodies was calculated for each measurement at the time of birth. A transfer efficiency of 1 indicates equal levels of antibodies in maternal and fetal circulation, while a transfer efficiency of greater than 1 indicates that the antibodies are being selectively transferred to the cord. For most measurements, no difference was observed in transfer efficiencies between vaccinated and placebo groups (Figure 2M), indicating that the overall antibody increases in the cord blood are the direct result of increased antibody in the maternal circulation. Surprisingly, significant decreases in transfer efficiency were observed in the vaccinated group compared to placebo group for ADCP and antibody-mediated MIP-1β production by NK cells (Figure 2M), whereas for all other measurements, no significant differences in transfer efficiency between vaccinated and placebo pairs were observed (Figures 2M and S1G and S1H). This suggests that the placenta may have a maximal transfer capacity for these antibodies, and when maternal levels exceed the maximum transferrable by the placenta, no more antibody may be provided to the fetus, supported by maternal antibody levels as a significant covariate of cord levels (Table S2). Collectively, these data point to an overall shift in vaccine-induced antibody levels during pregnancy, with consistent trends across influenza vaccine antigens (Figure S2). This shift in antibodies leads to a concordant augmentation of functionally enhanced antibodies in neonates, governed partially in a quantitative manner by augmented antibody levels, but also driven by qualitative changes in FcRn binding and NK cell activating antibodies.

FCGR-binding antibodies are selectively enhanced in mothers and cords following vaccination

Given the broad improvement of antibody amounts, FcR binding capacity, and functional activation in response to vaccination, we next aimed to determine which humoral features were most significantly altered by vaccination. To avoid overfitting, an elastic net-least absolute shrinkage and selection operator (LASSO) feature selection was first utilized to minimize the features to only include the fewest features that explained the overall variance in the dataset. Feature reduction was then followed by partial least-squares discriminant analysis (PLSDA) to visualize the separation between groups (Ackerman et al., 2018). These analyses were initially run including all samples for which antibody subclass, FcR binding, and functional data were available (Figure 3). Secondly, the analysis was run with only samples for which HAI titer values were calculated (Figure S3), a subset of the initial sample set. Importantly, while there were no

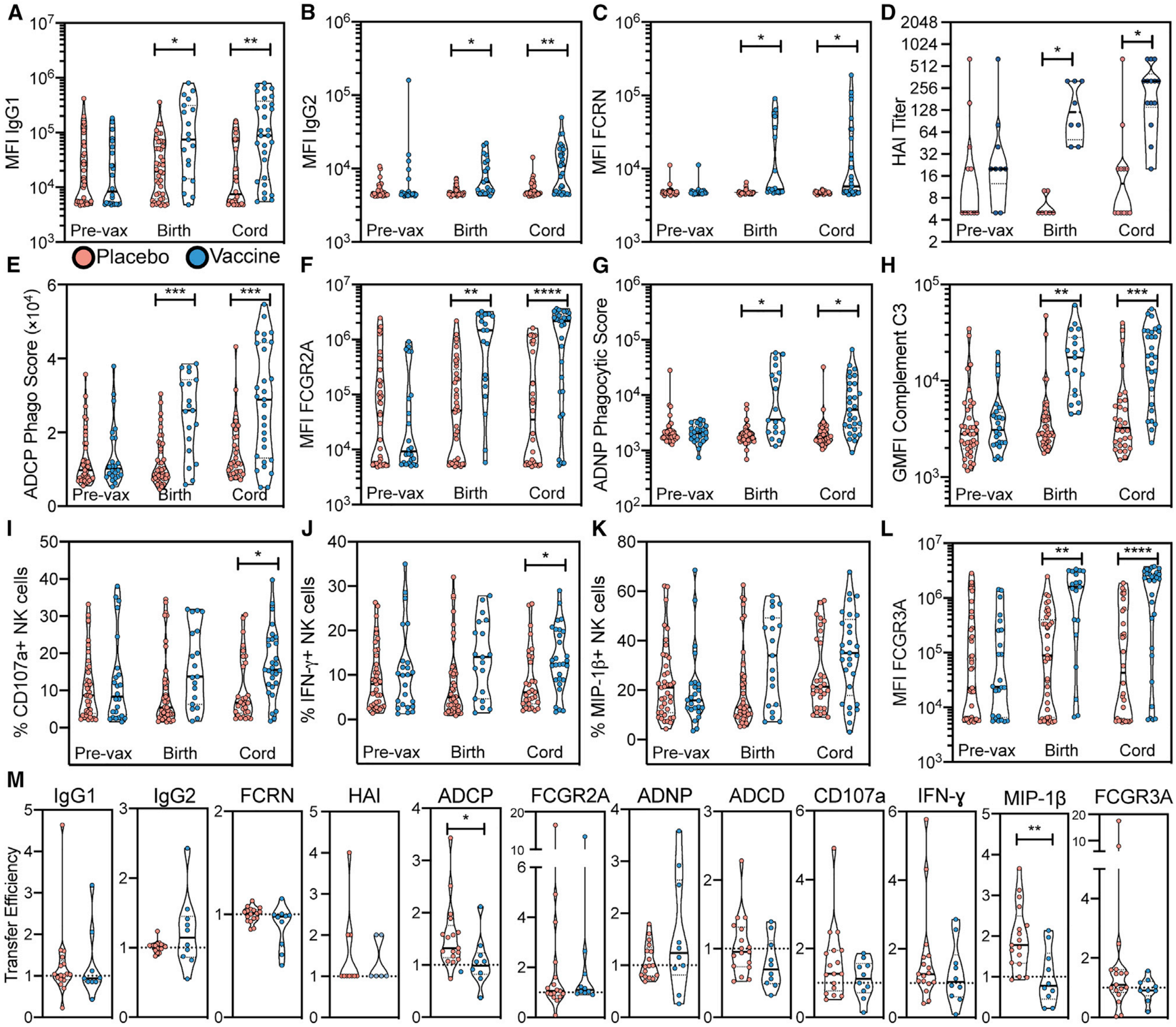

Figure 2. Vaccination boosts maternal and fetal antibodies but not transfer efficiency

Violin plots (A–L) show isotype amounts, FCGR binding levels, HAI titers, or functional levels of H1 A/California/07/2009-specific antibodies pre-vaccination in maternal circulation, at the time of birth in maternal circulation, and in the cord blood at the time of birth.

(A) H1 A/California/07/2009-specific IgG1 median fluorescence intensity (MFI) by Luminex bead-based assay.

(B) H1 A/California/07/2009-specific IgG2 MFI by Luminex bead-based assay.

(C) H1 A/California/07/2009-specific FCRN-binding antibody MFI by Luminex bead-based assay.

(D) HAI titer.

(E) Score of antibody-dependent cellular phagocytosis of immune complexed-H1 A/California/07/2009-coated beads.

(F) H1 A/California/07/2009-specific FCGR2A-binding antibody MFI by Luminex bead-based assay.

(G) Score of antibody-dependent neutrophil phagocytosis of immune complexed-H1 A/California/07/2009-coated beads.

(H) MFI of antibody-dependent C3 deposition on immune complexed-influenza HA-coated beads.

(I) HA-specific antibody-dependent CD107a expression by NK cells.

(J) HA-specific antibody-dependent IFN-y expression by NK cells.

(K) HA-specific antibody-dependent MIP-1b expression by NK cells.

(L and M) H1 A/California/07/2009-specific FCGR3A-binding antibody MFI by Luminex bead-based assay. Each dot represents an individual and violins show the distribution of the group. Red dots represent pairs where the mother received a placebo injection, and blue dots represent pairs where the mother received seasonal influenza vaccination. (Pre-vax placebo n = 39; pre-vax vaccinee n = 24; time of birth placebo n = 41; time of birth vaccinee n = 29; cord placebo n = 30; cord vaccine n = 29.) Significance was determined by mixed-effects analysis followed by Sidak's multiple comparisons test between placebo and vaccine groups within each time point. Violin plots (M) show transfer efficiencies calculated as the ratio between cord blood experimental value and maternal experimental value at time of birth for a given antibody isotype, FCGR-binding, or function. (Placebo pairs n = 17; vaccinee pairs n = 10.) Significance was determined by Mann-Whitney U test. *$p < 0.05$, **$p < 0.01$, ***$p < 0.001$, ****$p < 0.0001$. See also Figure S2.

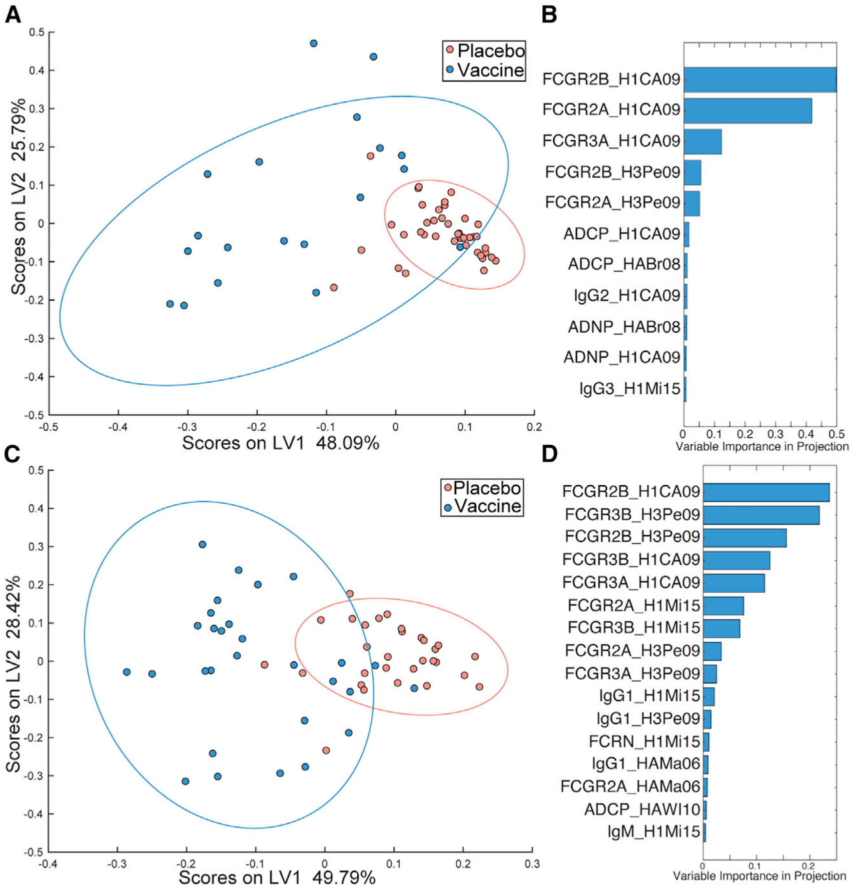

Figure 3. FCGR binding levels separate vaccinee mother-child pairs from placebo recipients

(A and C) Dot plots show partial least-squares discriminant analysis (PLSDA) scores along latent variable 1 (LV1) and latent variable 2 (LV2) for maternal samples at the time of birth (A) and cord blood samples (C). Models are significant (A: $p < 0.001$; C: $p < 0.01$) compared to permuted label models. PLSDA was modeled using least absolute shrinkage and selection operator (LASSO)-selected features out of the total pool of measured humoral biophysical and innate immune functional features. LV percentages represent the amount of total variance between samples captured by that LV. Bar plots show variable importance in protection (VIP) scores for the LASSO-selected features separating vaccinees from placebo recipients for maternal samples at the time of birth (B) and cord blood samples (D). VIP scores reflect the contribution of a feature across all latent variables. See also Figure S3.

differences between the mothers prior to vaccination, separation was observed across the placebo and vaccinated mothers after vaccination, at the time of birth (Figure 3A). Interestingly, this separation was not dependent on HAI, as models run on the subset of samples that had been profiled for HAI, the LASSO algorithm did not select HAI as a critical feature required to discriminate the mothers (Figures S3A and S3B), suggesting that vaccine-induced Fc profiles distinguished the groups more effectively. Specifically, using Fc features alone, vaccinated and unvaccinated mothers could be resolved with nearly 80% cross-validation accuracy, with model significance of $p < 0.001$ compared to permuted label model (Figure 3A). This separation was primarily driven by binding of both H1- and H3-specific antibodies to FCGRs, as summarized by the variable importance in projection (VIP) scores, which rank the minimal set of features required to discriminate the groups based on their relative importance in driving separation across the groups (Figure 3B). Additionally, phagocytic functions and levels of some H1-specific antibody IgG subclasses were enriched in the vaccinee serum compared to the placebo serum. While HAI was not selected by the model (Figure S3B), HAI was correlated with some model-selected features (Figure S3C). At the maternal time of birth, many of the FCGR-binding levels and antibody-dependent functions induced by vaccination were positively, although not significantly, correlated with HAI titers. This is consistent with the univariate analyses (Figure 2), highlighting vaccine-induced changes in antibody levels and HAI titers, but most significantly in FCGR-binding antibodies following vaccination.

Similar to maternal blood, robust separation was observed in HA-specific antibodies in cord blood using Fc profiling. Specifically, vaccinated and unvaccinated cord bloods could be discriminated with over 75% cross-validated accuracy using as few as 16 LASSO-selected features out of the overall 146 features measured for each sample ($p < 0.01$ compared to permuted label model; Figure 3C), and again, HAI titers were not selected as a discriminatory feature between vaccinated and placebo pairs (Figures S3D and S3E). Improved binding to multiple FCGRs was among the top features that distinguished cord antibodies from the infants of vaccinated and unvaccinated mothers (Figure 3D). Moreover, IgG1 levels were also included in the model, along with FcRn binding, suggesting both quantitative and qualitative features independently improved in the cord following vaccination (Figure 3D). Again, while HAI was not highlighted by the machine learning model (Figure S3E), HAI titers do correlate significantly with FCGR binding levels and HAI for both H1N1 and H3N2 influenza (Figure S3F). This increase in intercorrelation when compared to the maternal blood samples perhaps suggests a preferential transfer of antibodies capable of driving both neutralizing and extra-neutralizing antibody functions. These data illustrate the parallel shift in maternal (Figures 3A and 3B) and cord (Figures 3C and 3D) sera, highlighting the continuum across the mother and fetus resulting in the transfer of highly functional antibodies to the infant following vaccination.

FCGR-binding antibodies, rather than overall antibody levels, discriminate infants that later develop influenza infection

Follow-up of both vaccinated and unvaccinated mother-infant pairs allowed the identification of a number of infant influenza

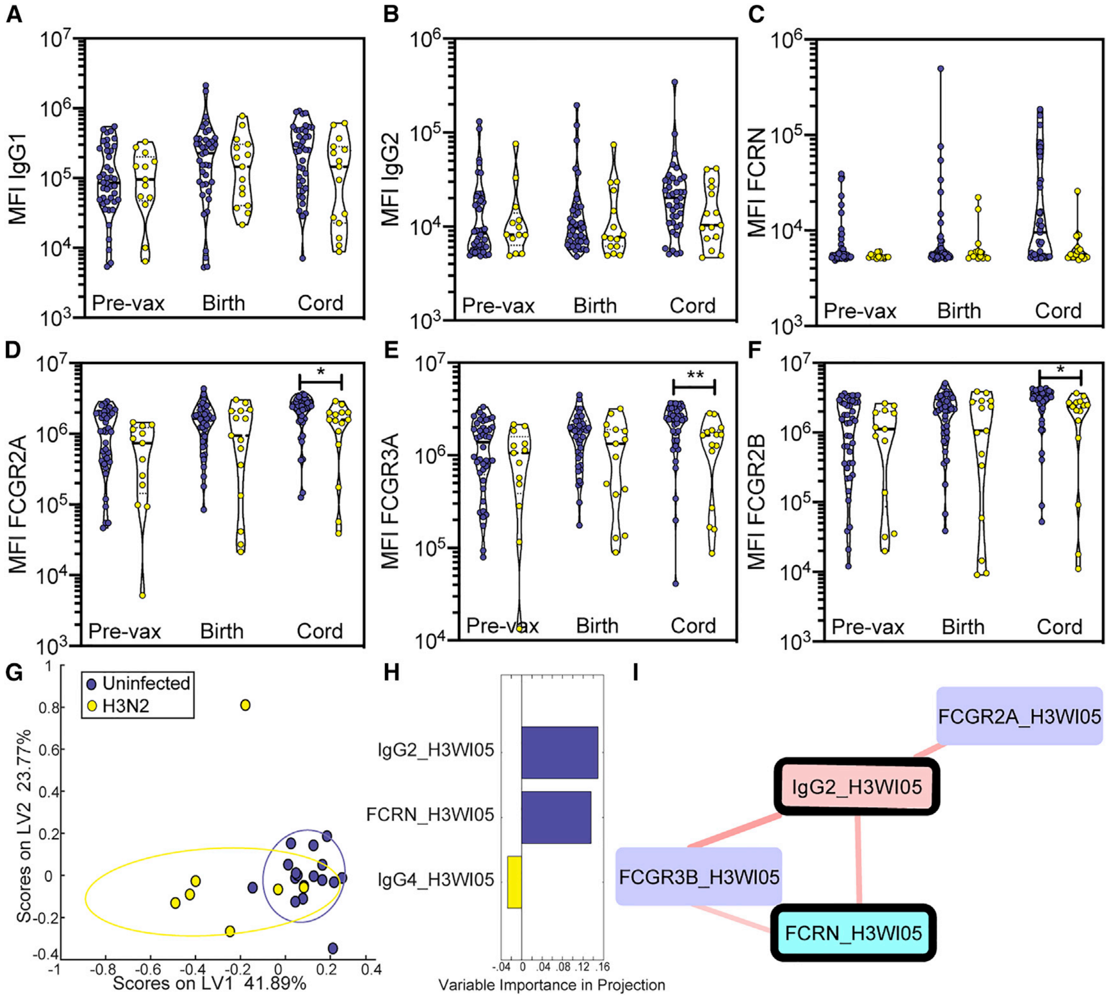

Figure 4. FCGR binding levels separate infected infants from uninfected infants

Violin plots (A–F) show isotype or FCGR binding levels of H3 A/Perth/16/2009-specific antibodies pre-vaccination, at the time of birth in maternal circulation, and in the cord blood at the time of birth.

(A) H3 A/Perth/16/2009-specific IgG1 MFI by Luminex bead-based assay.

(B) H3 A/Perth/16/2009-specific IgG2 MFI by Luminex bead-based assay.

(C) H3 A/Perth/16/2009-specific FCRN-binding antibody MFI by Luminex bead-based assay.

(D) H3 A/Perth/16/2009-specific FCGR2A-binding antibody MFI by Luminex bead-based assay.

(E) H3 A/Perth/16/2009-specific FCGR3A-binding antibody MFI by Luminex bead-based assay.

(F–I) H3 A/Perth/16/2009-specific FCGR3A-binding antibody MFI by Luminex bead-based assay. Each dot represents an individual and violins show the distribution of the group. Purple dots represent pairs where the infant would not be infected with influenza in the first six months of life, and yellow dots represent pairs where the infant would be infected with influenza A/H3N2 in the first six months of life. (Pre-vax control n = 42; pre-vax case n = 21; time of birth control n = 40; time of birth case n = 20; cord control n = 39; cord case n = 20.) Significance was determined by mixed-effects analysis followed by Sidak's multiple comparisons test between placebo and vaccine groups within each time point. $*p < 0.05$, $*p < 0.01$. Dot plot (G) shows PLSDA scores along latent variable 1 (LV1) and latent variable 2 (LV2) for transfer efficiencies. (Control pairs n = 17; case pairs n = 7.) PLSDA model (G) is significant compared to a permuted label model ($p = 0.02$). PLSDA was modeled using LASSO-selected features out of the total pool of H3-specific transfer efficiencies. Bar plot (H) shows VIP scores for the LASSO-selected features separating H3N2-infected infant transfer efficiencies from uninfected infant transfer efficiencies. Correlation network (I) shows positive correlation between LASSO-selected features (thick borders) and their co-correlates with a significance cutoff for inclusion at false discovery rate (FDR)-corrected q values < 0.05. Purple boxes indicate FCGRs, blue boxes indicate FCRN, and pink boxes indicate IgG amounts. See also Figure S4.

cases in this study (Katz et al., 2018). Here we aimed to determine whether antibody Fc profiles also tracked with differential infant outcomes after birth. Thus mother-infant pairs were divided into those who remained uninfected for the duration of this study and those who went on to have infant infection with H3N2 (Figures 1C and 1D). We focused on H3N2 infection as it was the most common strain of influenza responsible for infant infections in this study. No differences were observed in IgG1 and IgG2 levels across those with and without infant influenza infection (Figures 4A and 4B). Additionally, no differences were observed in the level of H3-specific FCRN-binding antibodies across the groups (Figure 4C). However, significant differences

in H3-specific FCGR binding capacity were observed in the cord blood of infants who later became infected with influenza compared with those who did not (Figures 4D–4F). Infants who would ultimately become infected had lower levels of FCGR-binding antibodies specific to the infecting strain of influenza when compared with infants who were not infected. These data point to the importance of qualitative changes in FCGR-binding antibodies transferred across the placenta, rather than the overall levels of HA-binding antibodies, in protection from influenza infection in early life.

To further dissect the minimal Fc features that were uniquely enriched in infants who did not develop influenza infection, we next used a similar LASSO-PLSDA model to our previous analysis (Figure 4G). Here, because of the differences in cord but not maternal antibodies, we focused on transfer ratios for all measured antibody Fc features across mother-infant pairs, comparing infants with and without H3N2 influenza infection at follow-up. Using these data, separation was achieved between the two groups, with nearly 80% cross-validation accuracy ($p = 0.02$). Interestingly, just three features were necessary to split the groups, including enhanced transfer of IgG2 and FcRn binding in the uninfected mother:cord pairs and elevated IgG4 transfer ratios in infected mother:cord pairs (Figure 4H). Enhanced transfer of H3-specific IgG2 and FcRn transfer efficiencies may mark overall enhanced transfer of H3-specific antibodies. Conversely, increased levels of H3-specific IgG4s, known to drive reduced FCGR activity (Bruhns et al., 2009), may mark dysregulated transfer of poorly functional antibodies in infants who went on to develop H3N2 infection.

Because LASSO does not select features based on their biological significance, but rather simply based on their overall influence on the variance across the sample groups, we next examined whether additional co-correlates existed that could provide information on the LASSO-selected features. In addition to IgG2 and FcRn transfer, transfer of H3-specific-FCGR2A- and FCGR3B-binding antibodies were positively correlated to the LASSO-selected features (Figure 4I). As a particular point of interest, the features selected in this analysis were not significantly correlated with HAI titer in these infants (Figure S4), indicating that extra-neutralizing antibody effector mechanisms may play a central part in preventing disease in infants. Overall, the results of this analysis point to IgG2 as a biomarker of more robust transfer of a protective immune response, resulting in the transfer of antibodies able to bind more effectively to FCGRs. These FCGR-binding antibodies may mechanistically underlie protection from influenza outcomes in neonates.

Cord blood from uninfected infants shows targeted humoral immune profile

Given the presence of both univariate and multivariate humoral discriminators across the infected and uninfected infants and their mothers, we next aimed to get a sense of the overall architecture of the polyclonal humoral immune response. Flower plots showing the relative magnitude of response within each group at each time point (Figure 5A) reveal overall higher humoral responses across influenza-specific antibody-dependent functions, antibody FCGR binding, and antibody isotype levels within the uninfected group as compared to the infected group at each time point. Levels of IgA were similar across the maternal groups and time points, emphasizing that current vaccination strategies do not induce robust IgA responses. Furthermore, expanded FCGR-binding profiles were observed among the uninfected maternal timepoints, but there was selective and more striking expansion of FCGR-binding antibodies in the cord blood of uninfected infants, linked to enhanced FcRn-binding antibodies in the cord, which indicate increased placental transfer. Specifically, we observed a selective enrichment of FCGR-binding antibodies specific to H1 CA/09 in the infected group both in maternal circulation at the time of birth and in cord blood (shown by the wedges marked with # in those plots). Since most of these infants were infected with H3N2, these data may point to a misdirected immune response primarily targeting H1N1 influenza, resulting in increased susceptibility to H3N2 influenza. In the uninfected group, the relative magnitude of the immune response was similar across all influenza strains (H1N1, H3N2, B).

To further dissect the humoral profile in the cord blood of infants, correlation matrices were calculated and visualized across functions, IgG1 levels, and FCGR-binding levels (Figure 5B). Focused and robust relationships between the same subtype of influenza and function were observed in uninfected infants, in contrast to the heterogeneous response patterns in the infected infant cord blood samples. In the uninfected infants (Figure 5B top), robust and highly focused correlations were observed between phagocytic functions and FCGR-binding antibodies to the same antigen specificity, with strong coordination within H1, H3, or B immune profiles. Infected infants exhibited a diffuse positive, but weaker, correlational profile marking a lower-level of functional coordination that may be insufficient to drive protective immunity (Figure 5B bottom). Within the sample group for which HAI titers were available, correlations were plotted between HAI and influenza-specific antibody-dependent innate immune functions, isotypes, and FCGR binding (Figure 5C). Here, increased correlation between neutralizing and extra-neutralizing functions/FCGR binding across isotypes was observed in the uninfected cord blood, whereas infected cord blood shows higher levels of strain-specific correlations. This further reinforces the conclusions drawn by the multivariate analyses in Figure 4 that protection tracks with FCGR-binding levels as well as the previously reported HAI titers (Katz et al., 2018). Thus, in addition to the individual biomarkers of protective humoral immunity, these data point to distinct humoral architecture in infants that ultimately are protected from influenza infection.

Vaccination shapes the transfer of protective immunity to the cord

Finally, we examined whether LASSO-selected features that distinguish vaccinee and placebo cord blood profiles are sufficient to predict protection from subsequent influenza infection. A model built on vaccine-augmented features classified infants into those that remained uninfected or became infected significantly better than a model using all features (Figures 6A and 6B). The vaccine-modified features were not uniformly associated with protection, but some features were enriched in the uninfected infants and others were enriched in the infected infants (Figure 6C). H3-specific FCGR responses were enriched in

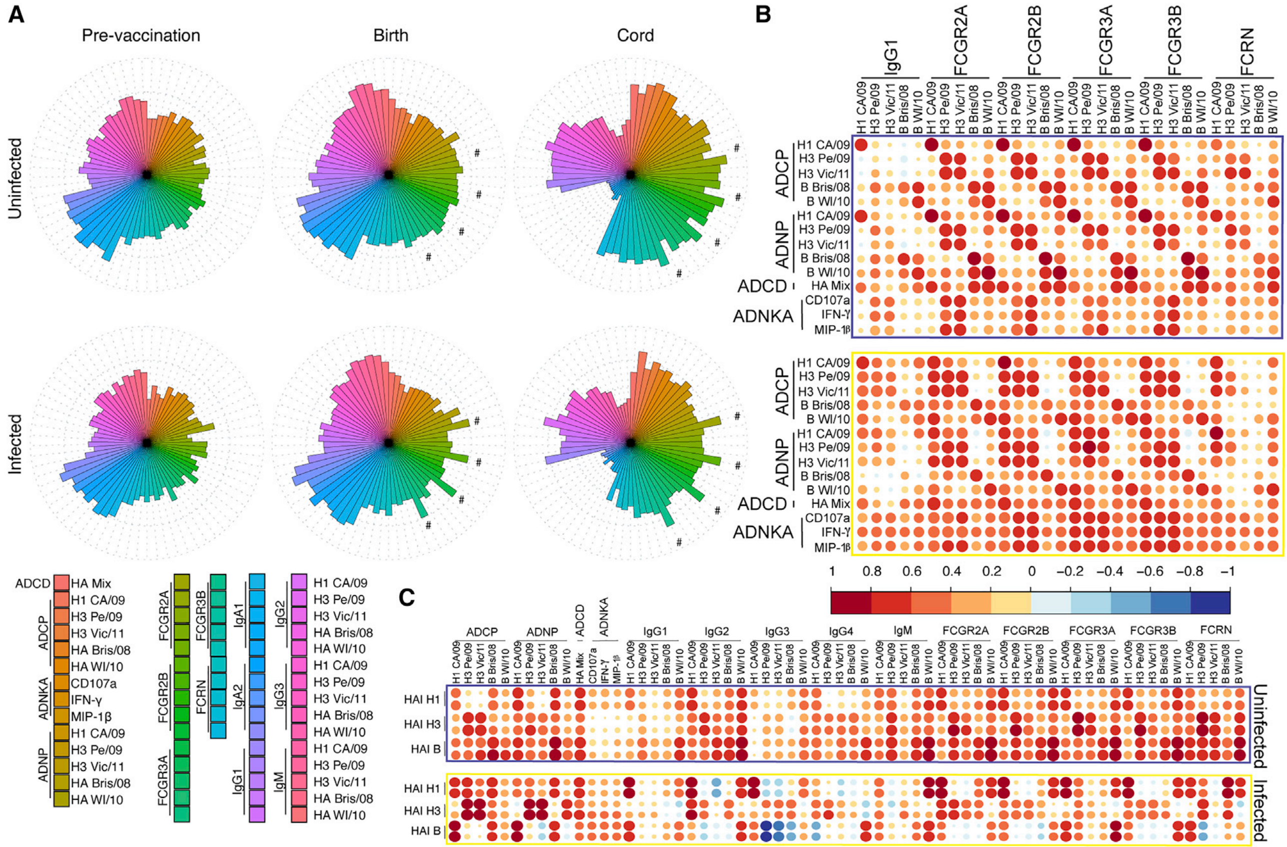

Figure 5. Humoral intercorrelation in cord blood differentiates infected infants from uninfected infants

(A–C) Flower plots (A) show average relative magnitude of measured humoral features within a time point (maternal pre-vaccination, maternal time of birth, and cord blood) and group (uninfected versus infected). # marks the wedges representing H1 CA/09 FCGR binding levels, as referenced in the text. Heat maps (B and C) show Spearman correlation coefficients for intercorrelations between measured antibody features. Within all features (except ADCD and antibody-dependent NK cell activation [ADNKA]) the five rows correspond to antigens specific for the five vaccine antigens: H1 A/California/07/2009, H3 A/Perth/16/2009, H3 A/Victoria/361/2011, B/Brisbane/06/2008, and B/Wisconsin/01/2010. ADCD and ADNKA were performed on pooled HA antigens, and ADNKA rows correspond to NK activation readouts: CD107a, IFN-γ, and MIP-1β. Matrices represent data for all cord blood samples pooled by infection outcome. For the subset of samples with HAI titers measured, matrix (C) represents Spearman correlation coefficients between HAI against vaccine strains (vertical) and antibody functional and biophysical features along the horizontal.

uninfected infants (Figure 6C), which was likely influential in protection given that dominant seasonal infections were H3N2 during the study period (Katz et al., 2018). Conversely, H1-specific FCGR responses were enriched in infected infants, supporting to the critical importance of strain-specific immunity in protection from disease. However, beyond IgG levels, HA strain-specific FCGR-binding features were highly discriminatory across the two groups when considered collectively in the latent variables of the PLSDA model (Figures 6C and S5). These data suggest that vaccine strategies able to tune FCGR binding in a strain-specific manner could potentially drive enhanced protection in the first months of life. The observation that the FCGR-binding levels of antibodies, but not overall amounts, were both enriched in cord blood by the vaccine (Figure 3) and involved in protection (Figures 4 and 6) suggests that the mechanism of protection by this vaccine may be driven by increased levels of the antibodies capable of recruiting FCGRs. Thus, beyond neutralizing antibody transfer from mother to child in the cord blood of vaccinated mothers (Katz et al., 2018), the data presented here point to a combined FCGR-binding antibody signature, across FCGR2A, FCGR2B, and FCGR3A specific to the infecting subtype of influenza, in vaccine-induced protection from influenza infection.

DISCUSSION

Beyond neutralization, mounting evidence points to a critical role of Fc effector functions in protection against influenza infection in adults (Boudreau and Alter, 2019; Vanderven and Kent, 2020). However, whether these responses are also critical for protection in infants, and whether they are tunable via influenza vaccination remain unclear. Using a unique cohort of longitudinally followed pregnant women who were enrolled in a randomized clinical trial of influenza vaccine or placebo, here we had a unique opportunity not only to probe the influence of vaccination on shaping Fc effector function across the maternal:fetal dyad,

CellPress
OPEN ACCESS

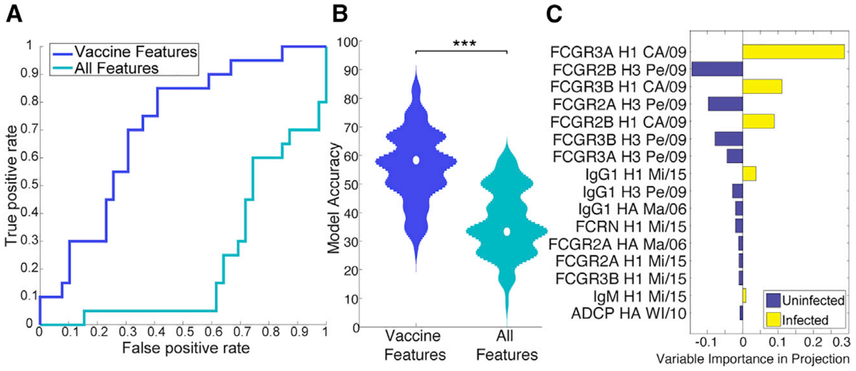

Figure 6. Vaccination strongly affects the protective humoral features in cord blood

(A–C) PLSDA models were trained to distinguish between cord blood of infants who go on to be infected versus those who do not, using either all measured features (light blue) or just those features determined to distinguish between cords of infants whose mothers were vaccinated and those who were not as shown in Figure 2B (dark blue). Receiver operating characteristic curves for final models are represented in (A). Positive rates refer to the model's ability to correctly classify cords into infected or uninfected: a false positive indicates an incorrect designation as a case, where a true positive indicates a correct designation as a case. Model accuracy in identifying cords of infected versus uninfected infants was tested for each fold- and replicate-specific test dataset. These values are represented in the violin plot (B). Significance was determined by Mann-Whitney U test (***$p < 0.001$). Bar plot (C) shows VIP scores for the vaccine LASSO-selected features separating infected infant transfer efficiencies from uninfected infant transfer efficiencies. Bars in yellow show features enriched in infected infants, while bars in purple show features enriched in uninfected infants.

but also to probe the antibody features that tracked with protection against infection. While naturally, irrespective of vaccination, the production of increased levels of FCGR and FcRn-binding antibodies occurred over the course of pregnancy, vaccination during pregnancy led to a robust augmentation of neutralizing and extra-neutralizing functional antibodies. Moreover, seasonal inactivated influenza vaccination preferentially increased the transfer of FCGR-binding antibodies across the placenta in coordination with the enrichment of both FCGR-binding and HAI titers in the cord blood. Yet most critically, lower levels of FCGR-binding antibody transfer and diffuse HA-specific functional antibody coordination were correlated with increased risk of infection independently of HAI titer. These data point to both a critical role for Fc effector function in protection from infection in early life, as well as opportunities for vaccination to selective augment functions that may be most desirable for protection.

Maternal vaccination against pertussis and influenza is protective for both pregnant individuals and neonates (Henkle et al., 2011; Madhi et al., 2014; Omer et al., 2015; Steinhoff et al., 2017; Switzer et al., 2019). Here we show that influenza vaccination in pregnancy increases Fc effector functions, highlighting that the quality of the humoral immune response increases with quantitative changes in vaccine-induced antibody amounts. Specifically, higher influenza-specific FCGR-binding levels were induced in mothers by influenza vaccination, leading to increased levels and protection in neonates. Further studies specifically designed to broadly capture HAI levels and deep systems serology across pregnant women will have power to fully dissect the impact of vaccination in shaping antibody Fab and Fc properties across the maternal:fetal dyad. Given that the placenta selectively transfers highly effective antibodies to the infant, developing vaccine strategies that can maximize the induction of functional antibodies in pregnancy may be key to improving neonatal immunity. Among proposed strategies, the use of adjuvants may bolster vaccine-induced immune responses (Boudreau et al., 2019; Nguyen-Contant et al., 2021) and could be used to selectively promote the most efficacious humoral immune responses. Future studies of adjuvanted influenza vaccination during pregnancy that characterize the functional humoral immune response would further elucidate these mechanisms and ensure that vaccines given during pregnancy are as efficacious as possible.

Previous studies in the elderly have shown mixed results as to the efficacy of seasonal influenza vaccines at eliciting FCGR-binding antibodies (Vanderven et al., 2017, 2020). However, elderly individuals are known to have decreased responses to traditional influenza vaccination strategies (Govaert et al., 1994; Gross et al., 1995). Vaccination during pregnancy does increase both HAI titers and influenza-specific IgG concentrations (Amin et al., 2020; Zhong et al., 2019), but the impact of pregnancy on the functional humoral immune response to influenza vaccination is only beginning to be understood. As shown in the primary results from the clinical trial (Katz et al., 2018), maternal influenza vaccination was linked to protection in infants that could not be fully explained by overall antibody transfer. While other factors, such as antibodies in breastmilk (Demers-Mathieu et al., 2019; Schlaudecker et al., 2013) and decreased exposure risk from a vaccinated mother likely also contribute to this protection and will require further study, here, vaccine impacts on the influenza-specific immune response, beyond antibody neutralization or hemagglutination inhibition titers, were identified. Influenza vaccination enriched the serum of both mothers and infants to induce more antibody-dependent innate immune functions and increased influenza-specific IgGs overall. Vaccinees and their infants were distinguished from placebo recipients by an enrichment of FCGR-binding antibodies. Moreover, the transplacental transfer from maternal to cord blood of these vaccine-induced FCGR-binding antibodies was linked to protection against influenza infection in infants. In sum, these functionally efficient antibodies are enriched by maternal vaccination and, when transferred to the infant, appear to be providing protection after birth. However, while this biomarker signature tracks with protection in this study, this finding will require validation in the future pursuit of defined correlates of neonatal immunity against influenza. In sum, the data presented here provide a critical new dimension to our understanding of maternal

influenza vaccination, highlighting the presence of functionally potent antibodies that can be boosted by vaccination during pregnancy and transported to the infants, resulting in protection of the infants from influenza infection. Which specific mechanisms are most critical for neonatal influenza infection or disease remain to be determined, as well as efforts to define the specific vaccine strategies that may selectively elicit these functions to protect this vulnerable population.

Limitations of the study

In this study, participants were under weekly respiratory virus symptom surveillance from enrollment to six months after birth, which captured symptomatic infections but did not track disease severity and may have missed asymptomatic infections or subclinical disease. While we cannot fully determine whether the uninfected dyads were exposed, clear deficits and differences were observed in antibody transfer across the dyads. However, to reduce variability in this study, we elected to focus solely on the dyads that became infected with H3N2, given the predominance of infections with this strain throughout the study. Moreover, due to the limited number of dyads infected with non-H3N2 strains, the power was insufficient to perform a secondary analysis. With future studies, focused on validation across years, it may be possible to determine whether the humoral features linked to protection here apply to additional influenza strains or other geographically distinct cohorts, and to further analyze the interplay of HAI with extra-neutralizing antibody functions, which was outside the scope of the current study. Finally, comorbidities, malnutrition, and coinfections may alter both vaccine-induced immunity in mothers, as well as Fc-placental transfer and correlates of immunity, confounding issues that will be of critical interest in countries such as Nepal, which has high rates of diarrheal diseases (Shakya, 2018), tuberculosis (Basnet et al., 2009), and child malnutrition (Katz et al., 2017). We note that Nepal also has a low prevalence of HIV (Cousins, 2018) and malaria (Gautam et al., 2019), which have been shown to diminish transplacental antibody transfer.

STAR★METHODS

Detailed methods are provided in the online version of this paper and include the following:

- KEY RESOURCES TABLE
- RESOURCE AVAILABILITY
  - Lead contact
  - Materials availability
  - Data and code availability
- EXPERIMENTAL MODEL AND SUBJECT DETAILS
  - Human subjects
  - Cell lines
- METHOD DETAILS
  - Ig subclassing/isotyping and FcR binding
  - Antibody-dependent cellular phagocytosis (ADCP)
  - Antibody-dependent neutrophil phagocytosis (ADNP)
  - Antibody-dependent complement deposition (ADCD)
  - Antibody-dependent NK cell activation (ADNKA)
- QUANTIFICATION AND STATISTICAL ANALYSIS

SUPPLEMENTAL INFORMATION

Supplemental information can be found online at https://doi.org/10.1016/j.celrep.2022.110337.

ACKNOWLEDGMENTS

We would like to thank Kira Newman for assistance in sample selection and Corinne Luedemann and Madeline Davis for administrative support. This work was supported by the following: the Ragon Institute, the Samana Cay Massachusetts General Hospital scholar program, and NIH grant 5R37AI080289, as well as NIH T32 AI007245 (to C.M.B.). We would also like to acknowledge Harvard CFAR for ongoing support through P30 AI060354-02.

AUTHOR CONTRIBUTIONS

C.M.B., J.A.E., H.Y.C., and G.A. designed the research study and wrote the manuscript. J.K., J.T., S.K., and S.C.L. designed and led the original clinical sample from which samples were drawn. K.D.S. and C.W. provided study samples. C.M.B. and J.S.B. conducted experiments, acquired data, and analyzed data. All authors contributed to the final version of the manuscript.

DECLARATION OF INTERESTS

G.A. is a founder of SeromYx Systems, Inc.

Received: April 29, 2021
Revised: October 19, 2021
Accepted: January 13, 2022
Published: February 8, 2022

REFERENCES

Ackerman, M.E., Moldt, B., Wyatt, R.T., Dugast, A.S., McAndrew, E., Tsoukas, S., Jost, S., Berger, C.T., Sciaranghella, G., Liu, Q., et al. (2011). A robust, high-throughput assay to determine the phagocytic activity of clinical antibody samples. J. Immunol. Methods *366*, 8–19.

Ackerman, M.E., Das, J., Pittala, S., Broge, T., Linde, C., Suscovich, T.J., Brown, E.P., Bradley, T., Natarajan, H., Lin, S., et al. (2018). Route of immunization defines multiple mechanisms of vaccine-mediated protection against SIV. Nat. Med. *24*, 1590–1598.

Akbulut, H., Çelik, A., Akbulut, A., and Ayar, A. (2012). Placental transfer of total IgG and IgG subclasses in a Turkis population living in eastern Anatolia. Nobel Med. *8*, 59–64.

Amin, A.B., Nunes, M.C., Tapia, M.D., Madhi, S.A., Cutland, C.L., Wairagkar, N., and Omer, S.B. (2020). Immunogenicity of influenza vaccines administered to pregnant women in randomized clinical trials in Mali and South Africa. Vaccine *38*, 6478–6483.

Basnet, R., Hinderaker, S.G., Enarson, D., Malla, P., and Mørkve, O. (2009). Delay in the diagnosis of tuberculosis in Nepal. BMC Public Health *9*, 1–5.

Boesch, A.W., Brown, E.P., Cheng, H.D., Ofori, M.O., Normandin, E., Nigrovic, P.A., Alter, G., and Ackerman, M.E. (2014). Highly parallel characterization of IgG Fc binding interactions. MAbs *6*, 915–927.

Boudreau, C.M., and Alter, G. (2019). Extra-neutralizing FcR-mediated antibody functions for a universal influenza vaccine. Front. Immunol. *10*, 1–14.

Boudreau, C.M., Yu, W., Suscovich, T.J., Talbot, H.K., Edwards, K.M., and Alter, G. (2019). Selective induction of antibody effector functional responses using MF59-adjuvanted vaccination. J. Clin. Invest. *130*, 662–672.

Brown, E.P., Licht, A.F., Dugast, A.S., Choi, I., Bailey-Kellogg, C., Alter, G., and Ackerman, M.E. (2012). High-throughput, multiplexed IgG subclassing of antigen-specific antibodies from clinical samples. J. Immunol. Methods *386*, 117–123.

Bruhns, P., Iannascoli, B., England, P., Mancardi, D.A., Fernandez, N., and Jorieux, S. (2009). Specificity and affinity of human Fc gamma receptors and

CellPress
OPEN ACCESS

their polymorphic variants for human IgG subclasses. Receptor *113*, 3716–3725.

Chung, A.W., Kumar, M.P., Arnold, K.B., Yu, W.H., Schoen, M.K., Dunphy, L.J., Suscovich, T.J., Frahm, N., Linde, C., Mahan, A.E., et al. (2015). Dissecting polyclonal vaccine-induced humoral immunity against HIV using systems serology. Cell *163*, 988–998.

Clements, T., Rice, T.F., Vamvakas, G., Barnett, S., Barnes, M., Donaldson, B., Jones, C.E., Kampmann, B., and Holder, B. (2020). Update on transplacental transfer of IgG subclasses: impact of maternal and fetal factors. Front. Immunol. *11*, 1–17.

Co, M.D.T., Cruz, J., Takeda, A., Ennis, F.A., and Terajima, M. (2012). Comparison of complement dependent lytic, hemagglutination inhibition and microneutralization antibody responses in influenza vaccinated individuals. Hum. Vaccin. Immunother. *8*, 1218–1222.

Costa-Carvalho, B.T., Vieira, H.M., Dimantas, R.B.R., Arslanian, C., Naspitz, C.K., Solé, D., and Carneiro-Sampaio, M.M.S. (1996). Transfer of IgG subclasses across placenta in term and preterm newborns. Braz. J. Med. Biol. Res. *29*, 201–204.

Cousins, S. (2018). Reaching Nepal's mobile population at risk of HIV. Lancet HIV *5*, e613–e614.

Dabrera, G., Zhao, H., Andrews, N., Begum, F., Green, H.K., Ellis, J., Elias, K., Donati, M., Zambon, M., and Pebody, R. (2014). Effectiveness of seasonal influenza vaccination during pregnancy in preventing influenza infection in infants, England, 2013/14. Eurosurveillance *19*, 1–4.

Dililo, D.J., Tan, G.S., Palese, P., and Ravetch, J.V. (2014). Broadly neutralizing hemagglutinin stalk – specific antibodies require Fc γ R interactions for protection against influenza virus in vivo. Nat. Med. *20*, 143–151.

Demers-Mathieu, V., Huston, R.K., Markell, A.M., Mcculley, E.A., Martin, R.L., and Dallas, D.C. (2019). Antenatal Influenza A-Specific IgA, IgM, and IgG Antibodies in Mother's Own Breast Milk and Donor Breast Milk, and Gastric Contents and Stools from Preterm Infants. Nutrients *11*, 1567.

DiLillo, D.J., Palese, P., Wilson, P.C., and Ravetch, J.V. (2016). Broadly neutralizing anti-influenza antibodies require Fc receptor engagement for in vivo protection. J. Clin. Invest. *126*, 605–610.

Doyle, T.J., Goodin, K., and Hamilton, J.J. (2013). Maternal and neonatal outcomes among pregnant women with 2009 pandemic influenza A(H1N1) illness in Florida, 2009-2010: a population-based cohort study. PLoS One *8*, e79040.

Englund, J.A., Mbawuike, I.N., Hammill, H., Hollemann, M.C., Baxter, B.D., and Glezen, W.P. (1993). Maternal immunization with influenza or tetanus toxoid vaccine for passive antibody protection in young infants. J. Infect. Dis. *168*, 647–656.

Fischinger, S., Fallon, J.K., Michell, A.R., Broge, T., Suscovich, T.J., Streeck, H., and Alter, G. (2019). A high-throughput, bead-based, antigen-specific assay to assess the ability of antibodies to induce complement activation. J. Immunol. Methods *473*, 112630.

Gautam, N., Kakchapati, S., Shrestha, S., and Wanishsakpong, W. (2019). Patterns and trends of malaria in 25 risk districts of Nepal from 2001 to 2017. Clin. Exp. Vaccin. Res. *8*, 77–85.

Govaert, T.M.E., Thijs, C.T.M.C.N., Masurel, N., Sprenger, M.J.W., Dinant, J., and Knottnerus, J.A. (1994). The efficacy of influenza vaccination in elderly individuals. A randomized double-blind placebo-controlled trial. J. Am. Med. Assoc. *272*, 1661–1665.

Gross, P.A., Hermogenes, A.W., Sacks, H.S., Lau, J., and Levandowski, R.A. (1995). The efficacy of influenza vaccine in elderly persons. A meta-analysis and review of the literature. Ann. Intern. Med. *123*, 518–527.

El Guerche-Séblain, C., Moureau, A., Schiffler, C., Dupuy, M., Pepin, S., Samson, S.I., Vanhems, P., and Schellevis, F. (2019). Epidemiology and burden of influenza in healthy children aged 6 to 35 months: analysis of data from the placebo arm of a phase III efficacy trial. BMC Infect. Dis. *19*, 4–9.

Hashira, S., Okitsu-Negishi, S., and Yoshino, K. (2000). Placental transfer of IgG subclasses in a Japanese population. Pediatr. Int. *42*, 337–342.

He, W., Chen, C.J., Mullarkey, C.E., Hamilton, J.R., Wong, C.K., Leon, P.E., Uccellini, M.B., Chromikova, V., Henry, C., Hoffman, K.W., et al. (2017). Alveolar macrophages are critical for broadly-reactive antibody-mediated protection against influenza A virus in mice. Nat. Commun. *8*, 1–13.

Henkle, E., Steinhoff, M.C., Omer, S.B., Roy, E., Arifeen, S.E., Raqib, R., McNeal, M., Breiman, R.F., Moss, W.J., and Zaman, K. (2011). Incidence of influenza virus infection in early infancy: a prospective study in South Asia. Pediatr. Infect. Dis. J. *30*, 170–173.

Huber, V.C., Lynch, J.M., Bucher, D.J., Le, J., and Metzger, D.W. (2001). Fc receptor-mediated phagocytosis makes a significant contribution to clearance of influenza virus infections. J. Immunol. *166*, 7381–7388.

Jarvis, J.R., Dorey, R.B., Warricker, F.D.M., Alwan, N.A., and Jones, C.E. (2020). The effectiveness of influenza vaccination in pregnancy in relation to child health outcomes: systematic review and meta-analysis. Vaccine *38*, 1601–1613.

Jegaskanda, S. (2018). The potential role of Fc-receptor functions in the development of a universal influenza vaccine. Vaccines *6*, 27.

Jegaskanda, S., Laurie, K.L., Amarasena, T.H., Winnall, W.R., Kramski, M., De Rose, R., Barr, I.G., Brooks, A.G., Reading, P.C., and Kent, S.J. (2013a). Age-associated cross-reactive antibody-dependent cellular cytotoxicity toward 2009 pandemic influenza A virus subtype H1N1. J. Infect. Dis. *208*, 1051–1061.

Jegaskanda, S., Weinfurter, J.T., Friedrich, T.C., and Kent, S.J. (2013b). Antibody-dependent cellular cytotoxicity is associated with control of pandemic H1N1 influenza virus infection of Macaques. J. Virol. *87*, 5512–5522.

Jegaskanda, S., Vanderven, H.A., Wheatley, A.K., and Kent, S.J. (2017). Fc or not Fc; that is the question: antibody Fc-receptor interactions are key to universal influenza vaccine design. Hum. Vaccin. Immunother. *13*, 1288–1296.

Jennewein, M.F., Goldfarb, I., Dolatshahi, S., Cosgrove, C., Noelette, F.J., Krykbaeva, M., Das, J., Sarkar, A., Gorman, M.J., Fischinger, S., et al. (2019). Fc glycan-mediated regulation of placental antibody transfer. Cell *178*, 202–215.e14.

Karsten, C.B., Mehta, N., Shin, S.A., Diefenbach, T.J., Slein, M.D., Karpinski, W., Irvine, E.B., Broge, T., Suscovich, T.J., and Alter, G. (2019). A versatile high-throughput assay to characterize antibody-mediated neutrophil phagocytosis. J. Immunol. Methods *471*, 46–56.

Katz, J., Englund, J.A., Steinhoff, M.C., Khatry, S.K., Shrestha, L., Kuypers, J., Mullany, L.C., Chu, H.Y., LeClerq, S.C., Kozuki, N., et al. (2017). Nutritional status of infants at six months of age following maternal influenza immunization: a randomized placebo-controlled trial in rural Nepal. Vaccine *35*, 6743–6750.

Katz, J., Englund, J.A., Steinhoff, M.C., Khatry, S.K., Shrestha, L., Kuypers, J., Mullany, L.C., Chu, H.Y., Leclerq, S.C., Kozuki, N., et al. (2018). Impact of timing of influenza vaccination in pregnancy on transplacental antibody transfer, influenza incidence, and birth outcomes: a randomized trial in Rural Nepal. Clin. Infect. Dis. *67*, 334–340.

Kay, A.W., Bayless, N.L., Fukuyama, J., Aziz, N., Dekker, C.L., Mackey, S., Swan, G.E., Davis, M.M., and Blish, C.A. (2015). Pregnancy does not attenuate the antibody or plasmablast response to inactivated influenza vaccine. J. Infect. Dis. *212*, 861–870.

Kopf, M., Abel, B., Gallimore, A., Carroll, M., and Bachmann, M.F. (2002). Complement component C3 promotes T-cell priming and lung migration to control acute influenza virus infection. Nat. Med. *8*, 373–378.

Lu, L.L., Chung, A.W., Rosebrock, T.R., Ghebremichael, M., Yu, W.H., Grace, P.S., Schoen, M.K., Tafesse, F., Martin, C., Leung, V., et al. (2016). A functional role for antibodies in tuberculosis. Cell *167*, 433–443.e14.

Madhi, S.A., Cutland, C.L., Kuwanda, L., Weinberg, A., Hugo, A., Jones, S., Adrian, P.V., Van Niekerk, N., Treurnicht, F., Ortiz, J.R., et al. (2014). Influenza vaccination of pregnant women and protection of their infants. N. Engl. J. Med. *371*, 918–931.

Mullarkey, C.E., Bailey, M.J., Golubeva, D.A., Tan, G.S., Nachbagauer, R., He, W., Novakowski, K.E., Bowdish, D.M., Miller, M.S., and Palese, P. (2016). Broadly neutralizing hemagglutinin stalk-specific antibodies induce potent phagocytosis of immune complexes by neutrophils in an Fc-dependent manner. MBio *7*, 1–12.

Nair, H., Brooks, W.A., Katz, M., Roca, A., Berkley, J.A., Madhi, S.A., Simmerman, J.M., Gordon, A., Sato, M., Howie, S., et al. (2011). Global burden of

respiratory infections due to seasonal influenza in young children: a systematic review and meta-analysis. Lancet *378*, 1917–1930.

Neuzil, K.M., Reed, G.W., Mitchel, E.F., Simonsen, L., and Griffin, M.R. (1998). Impact of influenza on acute cardiopulmonary hospitalizations in pregnant women. Am. J. Epidemiol. *148*, 1094–1102.

Nguyen-Contant, P., Sangster, M.Y., and Topham, D.J. (2021). Squalene-based influenza vaccine adjuvants and their impact on the hemagglutinin-specific B cell response. Pathogens *10*, 355.

Nunes, M.C., and Madhi, S.A. (2018). Influenza vaccination during pregnancy for prevention of influenza confirmed illness in the infants: a systematic review and meta-analysis. Hum. Vaccin. Immunother. *14*, 758–766.

O'Brien, K.B., Morrison, T.E., Dundore, D.Y., Heise, M.T., and Schultz-Cherry, S. (2011). A protective role for complement C3 protein during pandemic 2009 H1N1 and H5N1 influenza a virus infection. PLoS ONE *6*, e17377.

Ohmit, S.E., Petrie, J.G., Cross, R.T., Johnson, E., and Monto, A.S. (2011). Influenza hemagglutination-inhibition antibody titer as a correlate of vaccine-induced protection. J. Infect. Dis. *204*, 1879–1885.

Omer, S.B. (2017). Maternal immunization. N. Engl. J. Med. *376*, 1256–1267.

Omer, S.B., Richards, J.L., Madhi, S.A., Tapia, M.D., Steinhoff, M.C., Aqil, A.R., Wairagkar, N., Blackwelder, W., Bresee, J., Coulibaly, F., et al. (2015). Three randomized trials of maternal influenza immunization in Mali, Nepal, and South Africa: methods and expectations. Vaccine *33*, 3801–3812.

Ortiz, J.R., Englund, J.A., and Neuzil, K.M. (2011). Influenza vaccine for pregnant women in resource-constrained countries: a review of the evidence to inform policy decisions. Vaccine *29*, 4439–4452.

Rattan, A., Pawar, S.D., Nawadkar, R., Kulkarni, N., Lal, G., Mullick, J., and Sahu, A. (2017). Synergy between the classical and alternative pathways of complement is essential for conferring effective protection against the pandemic influenza A(H1N1) 2009 virus infection. PLoS Pathog. *13*, e1006248.

Schlaudecker, E.P., Steinhoff, M.C., Omer, S.B., McNeal, M.M., Roy, E., Arifeen, S.E., Dodd, C.N., Raqib, R., Breiman, R.F., and Zaman, K. (2013). IgA and neutralizing antibodies to influenza A virus in human milk: a randomized trial of antenatal influenza immunization. PLoS ONE *8*, 6–13.

Shakya, A. (2018). Diarroheal diseases; still a public health problem? Heal. Prospect *10*, 79–80.

Simister, N.E., and Mostov, K.E. (1989). An Fc receptor structurally related to MHC class I antigens. Nature *337*, 184–187.

Simister, N.E., and Rees, A.R. (1985). Isolation and characterization of an Fc receptor from neonatal rat small intestine. Eur. J. Immunol. *15*, 733–738.

Stapleton, N.M., Andersen, J.T., Stemerding, A.M., Bjarnarson, S.P., Verheul, R.C., Gerritsen, J., Zhao, Y., Kleijer, M., Sandlie, I., De Haas, M., et al. (2011). Competition for FcRn-mediated transport gives rise to short half-life of human IgG3 and offers therapeutic potential. Nat. Commun. *2*, 1–9.

Steinhoff, M.C., Katz, J., Englund, J.A., Khatry, S.K., Shrestha, L., Kuypers, J., Stewart, L., Mullany, L.C., Chu, H.Y., LeClerq, S.C., et al. (2017). Year-round influenza immunisation during pregnancy in Nepal: a phase 4, randomised, placebo-controlled trial. Lancet Infect. Dis. *17*, 981–989.

Switzer, C., D'Heilly, C., and Macina, D. (2019). Immunological and Clinical Benefits of Maternal Immunization against Pertussis: A Systematic Review (Springer Healthcare).

Takeda, S., Hisano, M., Komano, J., Yamamoto, H., Sago, H., and Yamaguchi, K. (2015). Influenza vaccination during pregnancy and its usefulness to mothers and their young infants. J. Infect. Chemother. *21*, 238–246.

Tapia, M.D., Sow, S.O., Tamboura, B., Téguеté, I., Pasetti, M.F., Kodio, M., Onwuchekwa, U., Tennant, S.M., Blackwelder, W.C., Coulibaly, F., et al. (2016). Maternal immunisation with trivalent inactivated influenza vaccine for prevention of influenza in infants in Mali: a prospective, active-controlled, observer-blind, randomised phase 4 trial. Lancet Infect. Dis. *16*, 1026–1035.

Terajima, M., Co, M.D.T., Cruz, J., and Ennis, F.A. (2015). High antibody-dependent cellular cytotoxicity antibody titers to H5N1 and H7N9 avian influenza A viruses in healthy US adults and older children. J. Infect. Dis. *212*, 1052–1060.

Tielsch, J.M., Steinhoff, M., Katz, J., Englund, J.A., Kuypers, J., Khatry, S.K., Shrestha, L., and LeClerq, S.C. (2015). Designs of two randomized, community-based trials to assess the impact of influenza immunization during pregnancy on respiratory illness among pregnant women and their infants and reproductive outcomes in rural Nepal. BMC Pregnancy Childbirth *15*, 1–10.

USAID (2021). Nepal HIV/AIDS (U.S. Agency for International Development). https://www.usaid.gov/nepal/hivaids.

Vanderven, H.A., and Kent, S.J. (2020). The protective potential of Fc-mediated antibody functions against influenza virus and other viral pathogens. Immunol. Cell Biol. *98*, 253–263.

Vanderven, H.A., Jegaskanda, S., Wines, B.D., Hogarth, P.M., Carmuglia, S., Rockman, S., Chung, A.W., and Kent, S.J. (2017). Antibody-dependent cellular cytotoxicity responses to seasonal influenza vaccination in older adults. J. Infect. Dis. *217*, 12–23.

Vanderven, H.A., Barr, I., Reynaldi, A., Wheatley, A.K., Wines, B.D., Davenport, M.P., Hogarth, P.M., and Kent, S.J. (2020). Fc functional antibody responses to adjuvanted versus unadjuvanted seasonal influenza vaccination in community-dwelling older adults. Vaccine *38*, 2368–2377.

Wang, X., Li, Y., O'Brien, K.L., Madhi, S.A., Widdowson, M.A., Byass, P., Omer, S.B., Abbas, Q., Ali, A., Amu, A., et al. (2020). Global burden of respiratory infections associated with seasonal influenza in children under 5 years in 2018: a systematic review and modelling study. Lancet Glob. Heal. *8*, 497–510.

Wong, S.-S., Duan, S., DeBeauchamp, J., Zanin, M., Kercher, L., Sonnberg, S., Fabrizio, T., Jeevan, T., Crumpton, J.-C., Oshansky, C., et al. (2017). The immune correlates of protection for an avian influenza H5N1 vaccine in the ferret model using oil-in-water adjuvants. Sci. Rep. *7*, 44727.

World Health Organization. (2012). Vaccines against influenza WHO position paper - November 2012. Wkly. Epidemiol. Rec. *47*, 461–476.

Zaman, K., Roy, E., Arifeen, S.E., Rahman, M., Raqib, R., Wilson, E., Omer, S.B., Shahid, N.S., Breiman, R.F., and Steinhoff, M.C. (2008). Effectiveness of maternal influenza immunization in mothers and infants. N. Engl. J. Med. *359*, 1555–1564.

Zhong, Z., Haltalli, M., Holder, B., Rice, T., Donaldson, B., O'Driscoll, M., Le-Doare, K., Kampmann, B., and Tregoning, J.S. (2019). The impact of timing of maternal influenza immunization on infant antibody levels at birth. Clin. Exp. Immunol. *195*, 139–152.

STAR★METHODS

KEY RESOURCES TABLE

REAGENT or RESOURCE	SOURCE	IDENTIFIER
Antibodies		
Mouse anti-human IgG1 PE	Southern Biotech	Cat#9052-09; RRID: AB_2796621
Mouse anti-human IgG2 PE	Southern Biotech	Cat#9060-09; RRID: AB_2796635
Mouse anti-human IgG3 PE	Southern Biotech	Cat#9210-09; RRID: AB_2796701
Mouse anti-human IgG4 PE	Southern Biotech	Cat#9200-09; RRID: AB_2796693
Mouse anti-human IgG-Fc PE	Southern Biotech	Cat#9040-09; RRID: AB_2796601
Mouse anti-human IgA1 PE	Southern Biotech	Cat#9130-09; RRID: AB_2796656
Mouse anti-human IgA2 PE	Southern Biotech	Cat#9140-09; RRID: AB_2796664
Mouse anti-human IgM PE	Southern Biotech	Cat#9020-09; RRID: AB_2796577
Mouse anti-human CD66b Pacific Blue	BioLegend	Cat#305112; RRID: AB_2563294
Goat anti-guinea pig C3 FITC	MP Biomedical	Cat#0855371; RRID: AB_2334449
CD107a PE-Cy5	BD Biosciences	Cat#555802; RRID: AB_396136
CD56 PE-Cy7	BD Biosciences	Cat#557747; RRID: AB_396853
CD16 APC-Cy7	BD Biosciences	Cat#557758; RRID: AB_396864
CD3 Pacific Blue	BD Biosciences	Cat#558117; RRID: AB_397038
MIP-1β PE	BD Biosciences	Cat#550078; RRID: AB_393549
IFN-ɣ FITC	BD Biosciences	Cat#340449; RRID: AB_400425
Chemicals, peptides, and recombinant proteins		
H1 A/California/07/2009	Immune Technology	Cat#IT-003-SW12p
H3 A/Perth/16/2009	Immune Technology	Cat#IT-003-0045p
HA B/Brisbane/60/2008	Immune Technology	Cat#IT-003B3p
H3 A/Victoria/361/2011	Immune Technology	Cat#IT-003-00423p
HA B/Wisconsin/01/2010	Immune Technology	Cat#IT-003-B7p
H3 A/Singapore/INFIMH-16-0019/2016	Immune Technology	Cat#IT-003-00343p
HA B/Colorado/06/2017	Immune Technology	Cat#IT-003-b12dTMP
H1 A/New Caledonia/1999	Immune Technology	Cat#IT-003-001p
H3 A/Wisconsin/67/X-161/2005	Immune Technology	Cat#IT-003-0041p
HA B/Malaysia/2506/2004	Immune Technology	Cat#IT-003-0054p
EBOV GPdTM	Mayflower Bioscience	Cat#0501-016
Human IL-15 recombinant protein	ThermoFisher Scientific	Cat#BMS319
Golgistop	BD Biosciences	Cat#554724; RRID: AB_2869012
FIX & PERM Cell Permeabilization Kit	ThermoFisher Scientific	Cat#GAS004
Streptavidin-R-Phycoerythrin	Prozyme	Cat#PJ31S
Human FCGR2A (R)	Duke Human Vaccine Institute	N/A
Human FCGR2B	Duke Human Vaccine Institute	N/A
Human FCGR3A (V)	Duke Human Vaccine Institute	N/A
Human FCGR3B	Duke Human Vaccine Institute	N/A
Human FCRN	Duke Human Vaccine Institute	N/A
Lyophilized Guinea Pig Complement	Cedarlane	Cat#CL-4051
Brefeldin A	Sigma-Aldrich	Cat#B7651-5MG
Gelatin Veronal Buffer with Magnesium & Calcium	Boston Bioproducts	Cat#IBB-300
Critical commercial assays		
RosetteSep Human NK Cell Enrichment Kit	Stem Cell Technologies	Cat#15065
Experimental models: Cell lines		
THP-1 cells	ATCC	Cat#TIB-202; RRID: CVCL_0006

(*Continued on next page*)

Continued

REAGENT or RESOURCE	SOURCE	IDENTIFIER
Software and algorithms		
Forecyt	Sartorius (Intellicyt)	https://www.sartorius.com/en/products/flow-cytometry/flow-cytometry-software
GraphPad Prism	GraphPad	https://www.graphpad.com/
MATLAB	MathWorks	https://www.mathworks.com/products/matlab.html
R	R Project	https://www.r-project.org
Cytoscape	Cytoscape Consortium	https://cytoscape.org/
Other		
MagPlex Microspheres	Luminex Corp.	Cat#MC12001-01, MC12003-01, MC12005-01, MC10008-YY, MC10012-YY, MC10015-YY, MC10020-YY, MC102024-01, MC10026-YY, MC10044-YY, MC10045-YY, MC10055-YY
FluoSpheres Carboxylate-modified Microspheres, 1.0 μm, yellow-green fluorescent	ThermoFisher Scientific	Cat#F8776
FluoSpheres Carboxylate-modified Microspheres, 1.0 μm, red fluorescent	ThermoFisher Scientific	Cat#F8775

RESOURCE AVAILABILITY

Lead contact

Further information and requests for resources and reagents should be directed to and will be fulfilled by the lead contact, Galit Alter (galter@mgh.harvard.edu).

Materials availability

This study did not generate new unique reagents.

Data and code availability

Data to replicate machine learning analyses in Figures 3 and 4 are available in the supplemental information. All additional data reported in this paper will be shared by the lead contact upon request.

Original code for machine learning models used in Figures 3 and 4 is available in this paper's supplemental information.

Any additional information required to reanalyze the data reported in this paper is available from the lead contact upon request.

EXPERIMENTAL MODEL AND SUBJECT DETAILS

Human subjects

Serum samples from a community-based, placebo-controlled trial of influenza vaccination in the rural Sarlahi district of Nepal in 2011–2014 (Katz et al., 2018) drawn from pregnant study participants prior to vaccination, at the time of birth, and from the umbilical cord, were obtained for systems serology analysis. Informed consent was obtained from all subjects. HIV status and malaria incidence were not recorded in the original trial. The HIV prevalence in Nepal is quite low and the study participants do not fall into currently accepted high risk groups (MSM, IV drug users, and migrant workers going to/from India) for HIV incidence in Nepal (USAID, 2021). Saline placebo was used for those assigned to the placebo group and vaccinees received the Vaxigrip vaccine (Sanofi Pasteur Ltd.) (Tielsch et al., 2015). More details about the demographic data of these subjects can be found in Table S1. The primary outcomes of the original trial were maternal influenza-like-illness, laboratory-confirmed influenza in infants, and low birthweight (Katz et al., 2018; Omer et al., 2015; Tielsch et al., 2015). Multiple secondary outcomes, including preterm birth, maternal laboratory-confirmed influenza, and effect of timing of vaccination, have already been investigated (Katz et al., 2018; Omer et al., 2015; Tielsch et al., 2015). Subjects were actively followed weekly for 6 months post-partum for fever or respiratory symptoms to track influenza infection. We selected a convenience subset of mother-infant pairs with and without evidence of infant influenza infection from the vaccine and the placebo arms of the trial, and performed a 1:2 match of cases:controls for each time point by vaccine status, vaccine season, month, and year of birth, infant sex, and preterm birth. Cases were defined as mother-infant pairs with infant laboratory-confirmed influenza infection and controls were defined as mother-infant pairs without infant influenza infection. Serum sample sets from a total of 114 mother-child pairs were included in the analysis presented here (Figure 1). Within this study cohort, 23 infants were infected with H3N2, 2 infants were infected with H1N1, and 6 infants were infected with influenza B. In two dyad pairs with infant

influenza infection and one pair without infant influenza infection, maternal influenza infection was also documented during the study period. A smaller subset of these samples had HAI data available from a previous study (Katz et al., 2018) (Figure 1). HAI assays were used to test for antibodies to influenza antigens contained in the vaccines by a CLIA certified laboratory at Cincinnati Children's Hospital per standard protocols for evaluating immunogenicity in influenza vaccination. Laboratory staff were blinded to the vaccine assignments and timing of vaccine. The parent trial is registered at clinicaltrials.gov (NCT01034254). This study was approved by the Massachusetts General Hospital Institutional Review Board.

Cell lines

THP-1 cells (cell line isolated from a 1-year-old male) were grown in R10 medium (RPMI plus 10% fetal bovine serum, L-glutamine, and penicillin/streptomycin) supplemented with 0.01% β-mercaptoethanol at 37°C, seeded at 200,000 cells/mL and split prior to reaching 1,000,000 cells/mL.

METHOD DETAILS

Ig subclassing/isotyping and FcR binding

Antigen-specific antibody subclass/isotypes and FcR binding were determined using a high-throughput Luminex-based assay (Brown et al., 2012). Antigens included were: **H1 A/California/07/2009, H3 A/Perth/16/2009, HA B/Brisbane/60/2008, H3 A/Victoria/361/2011, HA B/Wisconsin/01/2010,** H3 A/Singapore/INFIMH-16-0019/2016, HA B/Colorado/06/2017, H1 A/New Caledonia/1999, H3 A/Wisconsin/67/X-161/2005, HA B/Malaysia/2506/2004, all provided from Immune Technology Corp., and EBOV GPdTM from Mayflower Bioscience, St. Louis, MO. Bolded strains indicate vaccine strains during the trial. Antigens were coupled to carboxylate-modified microspheres (Luminex Corp., Austin, TX) by covalent NHS-ester linkages via EDC (ThermoFisher) and Sulfo-NHS (ThermoFisher) per manufacturer's instructions. These antigen-coated microspheres were added to non-binding 384-well plates (Grenier Bio-One, Kremsmunster, Austria) at 1,000 beads per well (45 μL). Serum samples were heat inactivated at 56°C for 30 min and centrifuged to remove aggregates. They were then diluted 1:100 for IgG1, total IgG, and FcRs, and 1:10 for IgG2-4, IgA1-2, and IgM prior to incubation with beads. 5 μL of diluted serum samples were added and incubated with microspheres on a shaker overnight at 4°C. Microspheres were washed, and PE-conjugated anti-IgG1, -IgG2, -IgG3, -IgG4, -IgG, -IgM, -IgA1, or -IgA2 detection antibodies (Southern Biotech, Birmingham, AL) or biotinylated FcRs (Duke Human Vaccine Institute (Boesch et al., 2014)) conjugated to streptavidin-PE (Prozyme, Hayward, CA) were added for 1 h at room temperature. The microspheres were washed and read on an iQue Screener Plus (Sartorius). All samples were run in duplicate and correlation between duplicates is ensured. Standard reagents to calculate absolute concentrations were not available for all subclasses/isotypes/Fc-receptors, so data are reported as the median fluorescence intensity (MFI) of detection antibody for the average of two replicates.

Antibody-dependent cellular phagocytosis (ADCP)

THP-1 phagocytosis of HA-coated beads was performed as previously described (Ackerman et al., 2011). HA antigens (H1 A/California/07/2009, H3 A/Perth/16/2009, H3 A/Victoria/361/2011, HA B/Wisconsin/01/2010, and HA B/Brisbane/60/2008) were purchased from Immune Technologies Corp, New York, NY. Each recombinant antigen was biotinylated with EZ-link NHS-LC-LC Biotin per manufacturer's instructions (Thermo Fisher Scientific). Biotinylated protein was adsorbed onto 1 μm fluorescent neutravidin beads at a ratio of 10 μg of protein to 10 μL of beads (Invitrogen, Carlsbad, CA). 10 μL of antigen-coated beads were then incubated with equal volume of serum samples diluted 1:200 in PBS for 2 h at 37°C in 96-well plates. Unbound antibody was washed away, and THP-1 cells added and incubated at 37°C for 16 h, then fixed. Phagocytosis was measured by flow cytometry on an iQue Screener Plus (Intellicyt, Albuquerque, NM). Data are reported as phagocytic scores, calculated as the % of bead positive cells × geometric mean fluorescence intensity (GMFI)/1,000. Each experiment was performed in two independent replicates and correlation between replicates was ensured.

Antibody-dependent neutrophil phagocytosis (ADNP)

Primary human neutrophil phagocytsosis was performed as previously described (Karsten et al., 2019). HA-coated 1 μm fluorescent neutravidin beads were prepared and incubated with equal volume 1:50 diluted serum samples as in the ADCP assay. Primary human leukocytes were isolated from healthy donors using Ammonium-Chloride Potassium (ACK) lysis, then added to the opsonized beads and incubated at 37°C for 1 h. The cells were then stained with fluorescent anti-human CD66b (Biolegend, San Diego, CA) and fixed prior to analysis on an iQue Screener Plus. Data are reported as phagocytic scores, calculated as the % of bead positive CD66+ cells × GMFI/1,000. Each sample was assayed on two healthy PBMC donors and correlation between donors was ensured ($p < 0.0001$).

Antibody-dependent complement deposition (ADCD)

Serum samples were heat inactivated at 56°C for 30 min and centrifuged to remove aggregates. Bead-based antibody-dependent complement deposition was analyzed as previously described (Fischinger et al., 2019). The 5 HA molecules used for the ADCP and ADNP assays were pooled in equal amounts and adsorbed to 1 μm fluorescent neutravidin beads as in the ADCP assay. HA-coated beads were incubated with 1:25 diluted, heat inactivated serum samples for 2 h at 37°C. Lyophilized guinea pig complement (Cedarlane, Burlington, Canada) was resuspended in ice-cold water, then diluted 1:60 in veronal buffer with calcium, magnesium, and

gelatin (Boston Bioproducts, Ashland, MA). 150 μL diluted complement was added to the opsonized beads and incubated for 20 min at 37°C. Beads were then washed with 15 mM EDTA and stained with anti-guinea pig C3 (MP Biomedicals, Santa Ana, CA). Samples were washed and analyzed on an iQue Screener Plus. Each experiment was performed in two independent replicates and correlation between replicates was ensured. Data are reported as the GMFI of C3 antibody for the average of two replicates.

Antibody-dependent NK cell activation (ADNKA)

Antibody-dependent NK cell activation and degranulation were measured as previously described (Jegaskanda et al., 2013b; Lu et al., 2016). ELISA plates (ThermoFisher NUNC MaxiSorp) were coated with pooled HAs (as in ADCD), then blocked. 50 μL of 1:25 diluted serum sample was added to each well and incubated for 2 h at 37°C. NK cells were isolated from buffy coats from healthy donors using the RosetteSep NK cell enrichment kit (STEMCELL Technologies, Vancouver, Canada) and rested in 1 ng/mL IL-15 at 37°C until needed. NK cells, with anti-CD107a PE-Cy5 (BD), brefeldin A (Sigma-Aldrich, St. Louis, MO), and GolgiStop (BD), were added and incubated for 5 h at 37°C. Cells were stained for surface markers using anti-CD56 PE-Cy7 (BD), anti-CD16 APC-Cy7 (BD), and anti-CD3 PacBlue (BD), then fixed and permeabilized using FIX & PERM Cell Permeabilization Kit (ThermoFisher). Cells were stained for intracellular markers using anti-MIP-1β PE (BD) and anti-IFNγ FITC (BD). Fixed cells were analyzed by flow cytometry on an iQue Screener Plus. NK cells were defined as CD3-and CD16/56+. Each sample was assayed on two healthy NK cell donors and correlation between donors was ensured ($p < 0.0001$). Further, each donor was independently quality controlled to ensure that positive control values were at least two standard deviations above negative control values. Data are reported as the percentage of cells positive for each marker (CD107a, IFN-ɣ, and MIP-1β).

QUANTIFICATION AND STATISTICAL ANALYSIS

Transfer ratios were calculated by dividing the levels of cord blood antibodies by the levels of maternal antibodies. One major outlier was removed from the FCGR3A vaccinee group for visualization purposes. Missing datapoints for mother:child pairs were not imputed for univariate analyses. Numbers of samples in each group analyzed can be found in Figure 1. All univariate statistical analyses were performed using GraphPad Prism. Statistical analyses for specific assays are detailed in the figure legends. Flower plots in Figure 5 were generated in RStudio version 3.5.1 on z-scored and percentile-ranked data using the package ggplot2. Correlation matrices in Figure 5 were generated in Rstudio version 3.5.1 using the package corrplot.

Classification models were developed in MATLAB (Code S1, S2, S3, S4, and S5). Missing values due to experimental error were imputed using k-nearest neighbor; data points were not imputed where samples did not exist in the analysis. Data were normalized using z scoring. Models were built using a previously published approach (Ackerman et al., 2018). Briefly, Elastic Net least absolute shrinkage and selection operator (LASSO) was used to select the fewest number of features to create separation between the groups. This modeling approach was evaluated through repeated fivefold cross validation, and fold-specific support vector machine (SVM) classifier model was trained using the LASSO-selected features and training data. LASSO was performed in a fivefold cross validation framework, meaning that for each fold 4/5 of the data were used for training and 1/5 of the data were used to test, with each fifth serving as the test set once. Data were visualized using a partial least squares discriminant analysis (PLSDA). The predictive model significance was measured using a null control model with permuted labels where features were not altered, which preserves the correlation structure within the data. Each control model was repeated a hundred times to generate the distribution of model accuracies and tested against the predictive power of the actual model. The p value represents the tail probability of the true classification accuracy in the distribution of control model accuracies. For the vaccination models, all measured features were input to the model. For the H3N2 infection models, features of H3-specific antibodies were input to the model. Correlation networks were visualized in Cytoscape with correlations calculated in MATLAB. Pairwise Spearman R values describe the correlation strength and false discovery rate (FDR)-corrected q values < 0.05 were used as a significance cutoff for inclusion in the correlation network.

Rank-based ANCOVA models (Table S2) were developed in MATLAB. Data for each Fc feature for samples with paired maternal time of birth and cord blood values ($n = 27$ pairs) were first ranked using the tiedrank2 function (https://www.mathworks.com/matlabcentral/fileexchange/72399-tiedrank2), then ANCOVA models were developed using the aoctools function. Values for the significance of maternal antibody levels as a covariate were Bonferroni corrected following model generation.

Cell Reports, Volume *38*

Supplemental information

Dissecting Fc signatures of protection in neonates following maternal influenza vaccination in a placebo-controlled trial

Carolyn M. Boudreau, John S. Burke IV, Kiel D. Shuey, Caitlin Wolf, Joanne Katz, James Tielsch, Subarna Khatry, Steven C. LeClerq, Janet A. Englund, Helen Y. Chu, and Galit Alter

Figure S1. Vaccination boosts maternal and fetal antibodies, but not transfer efficacy, Related to Figure 2.

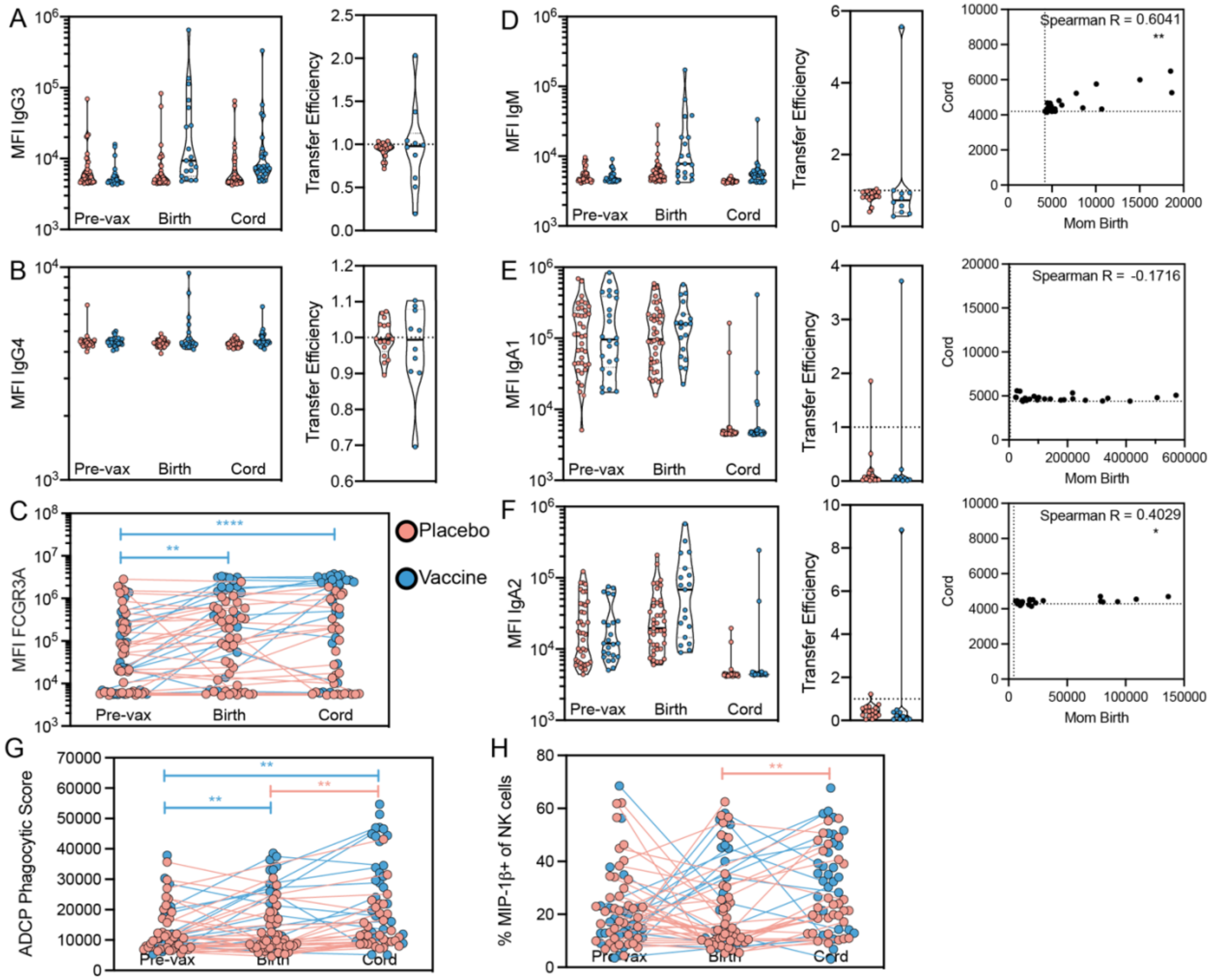

Paired violin plots show levels of H1 A/California/07/2009-specific antibodies pre-vaccination in maternal circulation, at the time of birth in maternal circulation, and in fetal circulation in the cord blood at the time of birth and the transfer efficiency of each type of antibody across the placenta at the time of birth. Each dot represents an individual and violins show the distribution of the group. Red dots represent pairs where the mother received placebo and blue dots represent pairs where the mother received influenza vaccination. Violin plots show transfer efficiencies calculated as the ratio between cord blood antibodies and maternal antibodies at time of birth. (**A**) H1-specific IgG3 by Luminex MFI; (**B**) H1-specific IgG4 by Luminex MFI; (**D**) H1-specific IgM by Luminex MFI; (**E**) H1-specific IgA1 by Luminex MFI; (**F**) H1-specific IgA2 by Luminex MFI. Dot plots (**D-F**) show correlation between H1 A/California/07/2009-specific isotype MFIs in maternal circulation at the time of birth (X axis) and in cord blood at the time of birth (Y axis). Each dot represents a matched mother-child pair. Dotted lines represent

the lower limit of detection of the assay. Spearman R values are reported with significance. Colored dot plots show change over time for connected maternal:fetal dyads. (**C**) H1-specific FCGR3A by Luminex MFI; (**G**) Phagocytosis by monocytes of H1-coated immune complexed beads; (**H**) MIP-1b expression by H1-specific antibody-stimulated NK cells. Significance was calculated by mixed effects analysis with Sidak's multiple comparisons test or Mann-Whitney U test as appropriate, * $p < 0.05$, ** $p < 0.01$, *** $p < 0.001$, **** $p < 0.0001$.

Figure S2. Vaccination boosts maternal and fetal antibodies specific to H3, Related to Figure 2

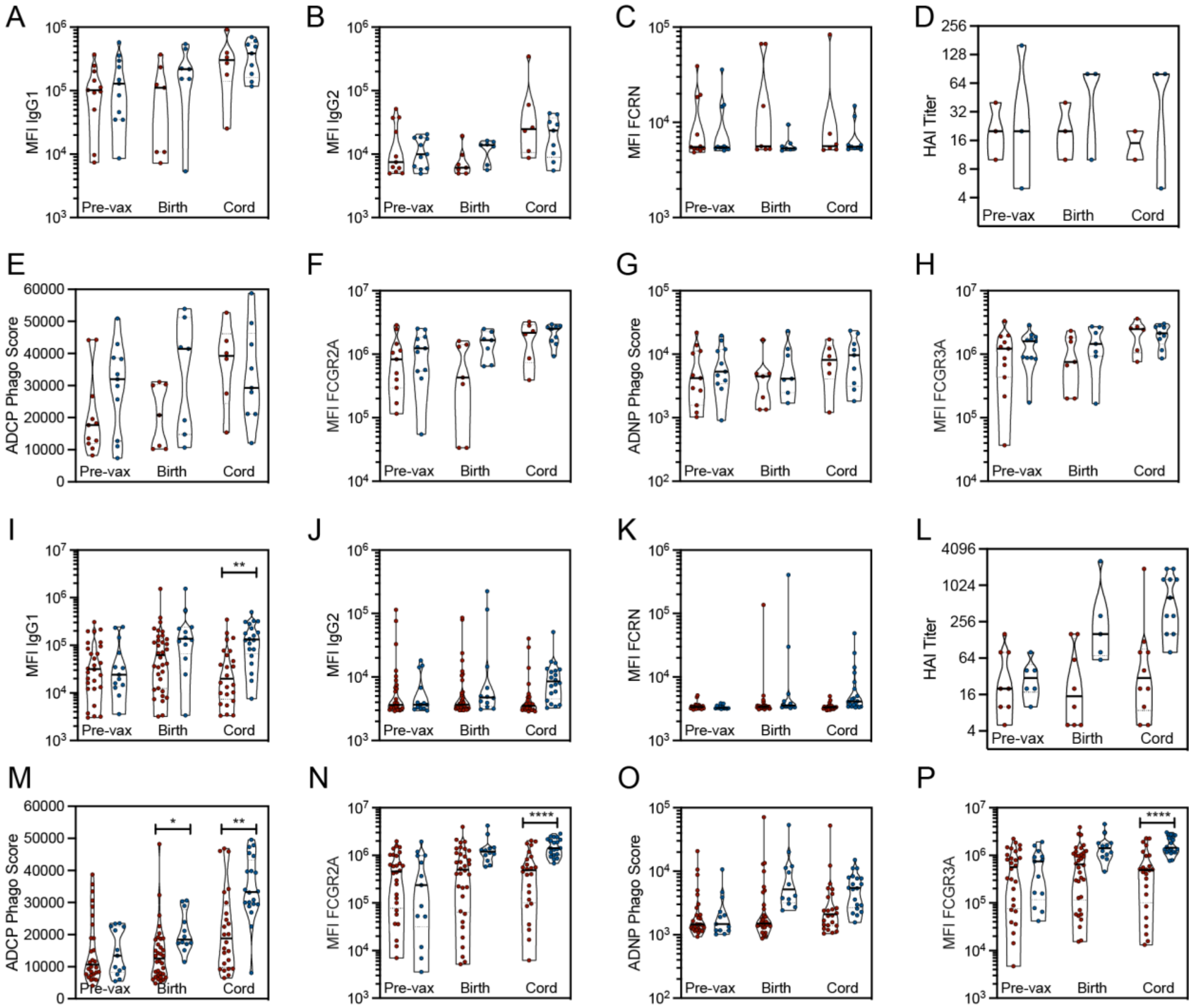

Paired violin plots show levels of (**A-H**) H3 A/Perth/16/2009-specific or (**I-P**) H3 A/Victoria/361/2011-specific antibodies pre-vaccination in maternal circulation, at the time of birth in maternal circulation, and in fetal circulation in the cord blood at the time of birth and the transfer efficiency of each type of antibody across the placenta at the time of birth. Each dot represents an individual and violins show the distribution of the group. Red dots represent pairs where the mother received placebo and blue dots represent pairs where the mother received influenza vaccination. (**A,I**) IgG1 by Luminex MFI; (**B,J**) IgG2 by Luminex MFI; (**C,K**) FCRN binding by Luminex MFI; (**D,L**) HAI titers; (**E,M**) cellular phagocytosis; (**F,N**) FCGR2A binding by Luminex MFI; (**G,O**) neutrophil phagocytosis; (**H,P**) FCGR3A binding by Luminex MFI. For (**A-C, E-H**) H3 A/Perth/16/2009-specific responses: pre-vax placebo n = 11, pre-vax vaccine n = 11,

time of birth placebo n = 7, time of birth vaccine n = 7, cord placebo n = 6, cord vaccine n = 9. For (**D**) H3 A/Perth/16/2009-specific HAI: pre-vax placebo n = 3, pre-vax vaccine n = 3, time of birth placebo n = 3, time of birth vaccine n = 3, cord placebo n = 2, cord vaccine n = 3. For (**I-K, M-P**) H3 A/Victoria/361/2011-specific responses: pre-vax placebo n = 28, pre-vax vaccine n = 13, time of birth placebo n = 34, time of birth vaccine n = 12, cord placebo n = 24, cord vaccine n = 20. For (**L**) H3 A/Victoria/361/2011-specific HAI: pre-vax placebo n = 8, pre-vax vaccine n = 6, time of birth placebo n = 8, time of birth vaccine n = 5, cord placebo n = 10, cord vaccine n = 11.

Figure S3. HAI is not a key feature separating placebo recipients and vaccinees but correlates with some key features, Related to Figure 3

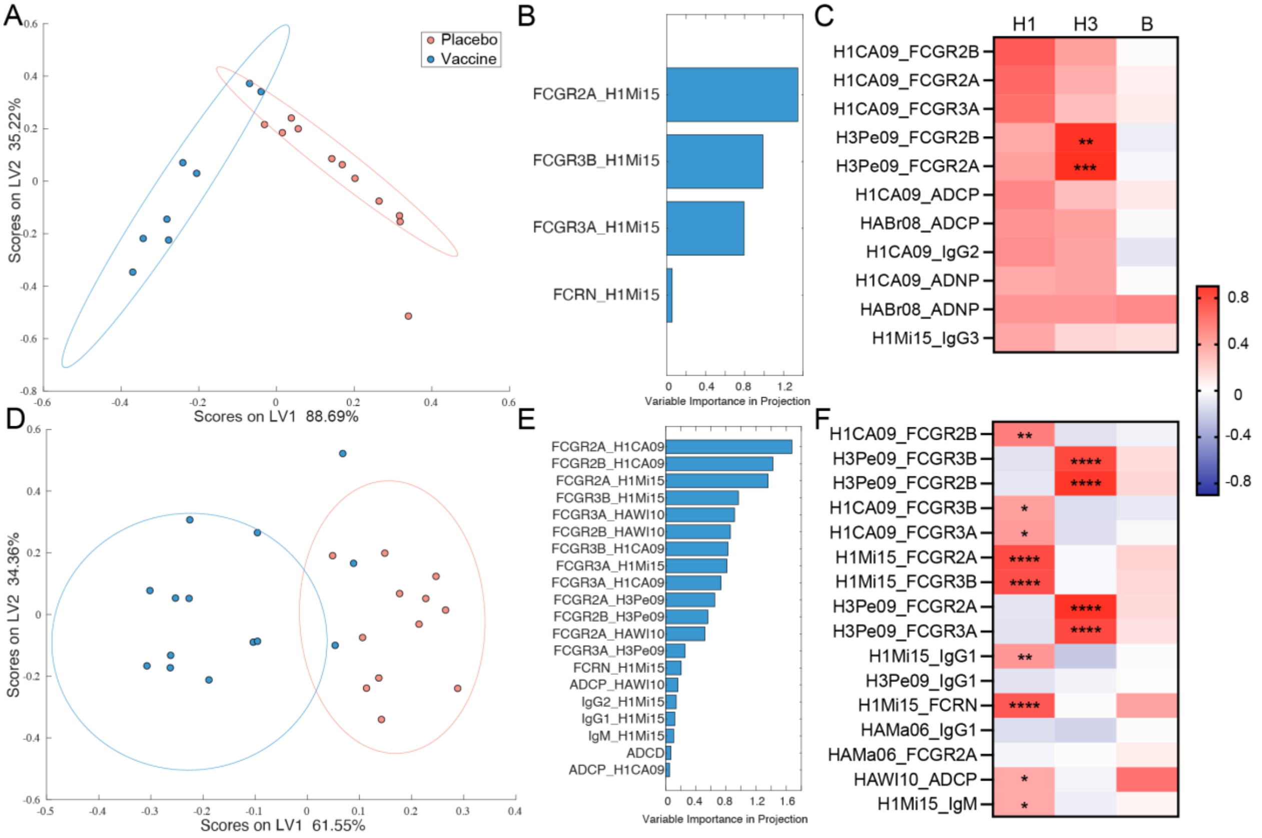

Dot plots show PLSDA scores along latent variable 1 (LV1) and latent variable 2 (LV2) for maternal samples at the time of birth (**A**, p < 0.001 compared to permuted label model) and cord blood samples (**D**, p < 0.01 compared to permuted label model). PLSDA was modeled using LASSO-selected features out of the total pool of measured humoral features, including HAI. LV percentages represent the amount of total variance between samples captured by that LV. Bar plots show VIP scores for the LASSO-selected features separating vaccinees from placebo recipients for maternal samples at the time of birth (**B**) and cord blood samples (**E**). VIP scores reflect the contribution of a feature across all latent variables. Correlation matrices show Spearman correlation R values calculated pairwise between HAI titers and LASSO-selected features for samples taken from maternal circulation (**C**) and cord blood (**F**) at the time of birth. Stars indicate Bonferroni-corrected p values: * < 0.05, ** < 0.01, *** < 0.001, **** <0.0001.

Figure S4. HAI titers do not significantly correlate with key features separating infected and uninfected infants, Related to Figure 4.

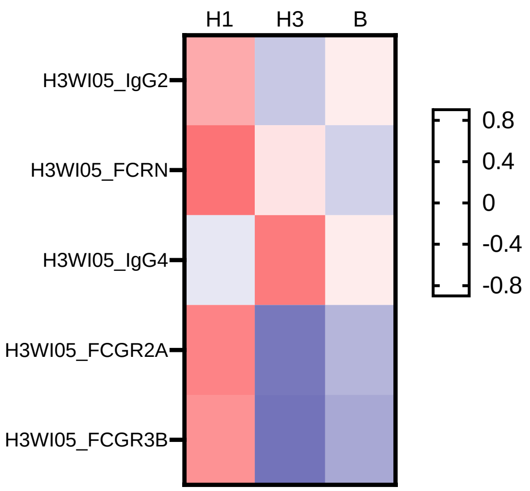

Correlation matrix shows Spearman correlation R values calculated pairwise between HAI titers and LASSO-selected features for samples cord blood at the time of birth for infants who went on to become infected with H3N2 influenza or remained uninfected throughout the trial. No correlations were significant when corrected for multiple comparisons.

Figure S5. Vaccine-induced humoral features are predictive of protection from influenza infection in infants, Related to Figure 6.

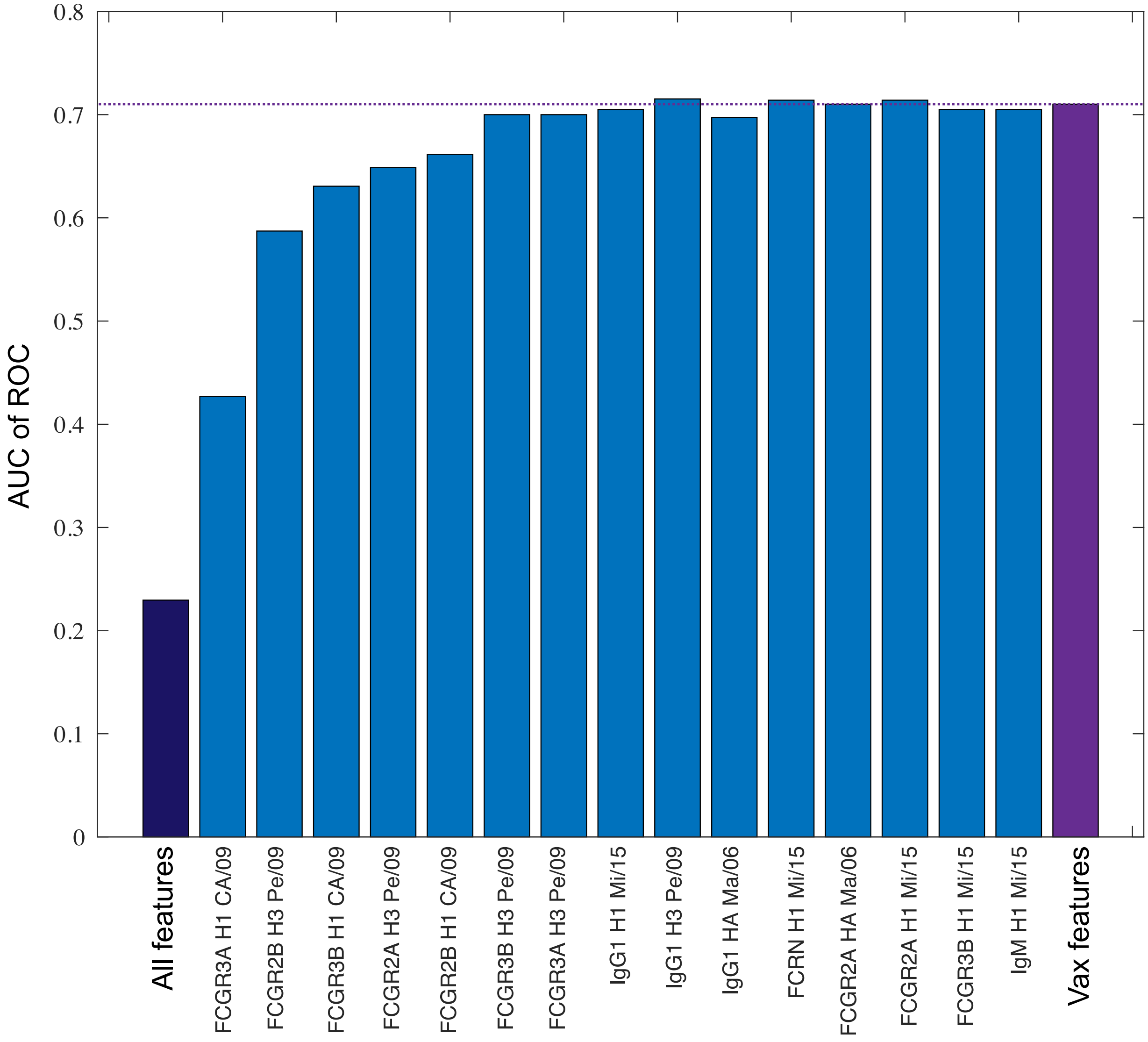

Bar plot shows the area under the curve for ROC of the PLSDA separating H3-infected infants from uninfected infants. Dark blue bar (left) shows AUC of ROC for all measured humoral features (as shown in Figure 6). Purple bar (right) shows AUC of ROC for vaccine LASSO-selected humoral features (as shown in Figure 6). Blue bars in between show sequential addition of individual features; leftmost blue bar shows only FCGR3A H1 CA/09, then each sequential feature is added moving from left to right.

Table S1. Demographic information about study participants, Related to STAR Methods

	Placebo	Vaccine	Control	Case
**Demographic Data Available**	60	49	77	32
**Missing Demographic Data**	1	0	0	1
**Male infants**	16.7% (10)	26.5% (13)	23.4% (18)	15.6% (5)
**Preterm infants**	28.3% (17)	20.4% (10)	23.4% (18)	28.1% (9)
**Vaccine Strains**				
A/CA/07/2009, A/Perth/16/2009, B/Brisbane/60/2008	23.3% (14)	30.6% (15)	28.6% (22)	21.9% (7)
A/CA/07/2009, A/Victoria/361/2011, B/Wisconsin/1/2010	76.7% (46)	69.4% (34)	71.4% (55)	78.1% (25)
**Vaccination Season**				
Dec-Feb	55% (33)	30.6% (15)	41.6% (32)	50% (16)
Mar-May	21.7% (13)	34.7% (17)	27.3% (21)	28.1% (9)
Jun-Aug	1.7% (1)	4.1% (2)	4.9% (3)	0% (0)
Sep-Nov	21.7% (13)	30.6% (15)	27.3% (21)	21.9% (7)
**Birth Season**				
Dec-Feb	18.3% (11)	40.8% (20)	32.5% (25)	18.8% (6)
Mar-May	48.3% (29)	14.3% (7)	29.9% (23)	40.6% (13)
Jun-Aug	31.7% (19)	32.7% (16)	32.5% (25)	31.3% (10)
Sep-Nov	1.7% (1)	12.2% (6)	5.2% (4)	9.4% (3)
**Approx. Gestational Age at Vaccination**				
1st Trimester	3.3% (2)	6.1% (3)	5.2% (4)	3.1% (1)
2nd Trimester	76.7% (46)	61.2% (30)	66.2% (51)	78.1% (25)
3rd Trimester	20% (12)	32.7% (16)	28.6% (22)	18.8% (6)

Table S2. Significance of covariates to vaccination across placebo and vaccine recipient pairs, related to Figure 2.

Measurement	Significance of Covariate (p)	Bonferroni corrected p	
IgG1	6.45E-06	7.09E-05	****
IgG2	1.29E-04	0.0014	**
FCRN	0.0465	0.5111	ns
ADCP	5.78E-05	6.36E-04	***
FCGR2A	9.32E-10	1.03E-08	****
ADNP	0.0013	0.0138	*
ADCD	7.53E-05	8.29E-04	***
ADNKA CD107a	2.91E-05	3.20E-04	***
ADNKA IFN-y	3.09E-04	0.0034	**
ADNKA MIP-1b	2.17E-04	0.0024	**
FCGR3A	1.41E-06	1.55E-05	****
